# Novel Meta-Diamide Compounds Containing Sulfide Derivatives Were Designed and Synthesized as Potential Pesticides

**DOI:** 10.3390/molecules29061337

**Published:** 2024-03-17

**Authors:** Jingwen Wu, Shuaihui Dang, Yan Zhang, Sha Zhou

**Affiliations:** 1 College of Resources and Environmental Engineering, Shanghai Polytechnic University, Shanghai 201209, China; 20221516055@stu.sspu.edu.cn (J.W.); 20221516008@stu.sspu.edu.cn (S.D.); 2 Collaborative Innovation Center of Zhejiang Green Pesticide, National Joint Local Engineering Laboratory for High-Efficient Preparation of Biopesticide, School of Forestry and Biotechnology, Zhejiang A & F University, Hangzhou 311300, China; 15822308335@163.com

**Keywords:** *m*-diamine, sulfide, synthesis, insecticidal activities

## Abstract

The meta-diamide (*m*-diamide) insecticide, Broflanilide, was characterized by its high efficiency, low toxicity and lack of cross-resistance with traditional GABA receptors. In accordance with the principles of drug molecular design, easily derivable sulfur with diverse bioactivities was introduced while leading with the parent Broflanilide. Twelve novel *m*-diamide target compounds containing sulfide derivatives were synthesized through exploration guided by the literature. Their structures were confirmed by melting points, ^1^H NMR, ^13^C NMR and HRMS. Insecticidal activity assessments revealed that most target compounds **A**–**D** exhibited 100% lethality against *Plutella xylostella* (*P. xylostella*) and *Aphis craccivora Koch* (*A. craccivora*) at 500 mg·L^−1^. Notably, for *P. xylostella*, compounds **C-2**, **C-3**, **C-4** and **D-2** demonstrated 60.00–100.00% insecticidal activity even at a concentration as low as 0.625 mg·L^−1^. As determined by structure–activity relationship (SAR) analysis, compounds with R_1_ = CH_3_ and R_2_ = Br (**B-1**, **C-2** and **D-2**) and sulfoxide compound **C-3** contained 100.00% lethality against *A. craccivora* at 500 mg·L^−1^, surpassing the lethality when leading with the parent Broflanilide in terms of efficacy. Consequently, it can be inferred that the sulfoxide compound (**C-3**) requires further investigation as a potential active molecule for new insecticides. These explorations provide valuable references for future research on the synthesis and insecticidal activities of sulfide-containing *m*-diamide compounds.

## 1. Introduction

**Figure 1:**
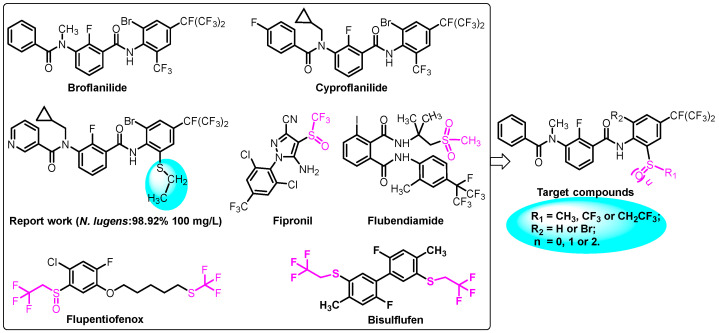
The structures of *m*-diamide, sulfide-containing drugs and the target compounds designed in this paper.

The continual discovery and development of novel pesticides with unique structures and superior biological activities remain paramount objectives for researchers in the field of agrochemicals [1,2]. Broflanilide [3] (BASF (Ludwigshafen, Germany) and Mitsui Chemicals, Inc. (Tokyo, Japan)) and Cyproflanilide [4] (Tahoe Group) (Figure 1) acting on *γ*-aminobutyric acid (GABA) receptors, which regulate the transmission of chloride ions into cells, causing pests to vomit and excite they are killed, are typical representatives of *m*-diamide insecticide. Their outstanding insecticidal activities and novel structures have quickly garnered significant interest among pesticide researchers. Recently, the Wu [5] group reported that compounds with sulfides introduced in the place of the trifluoromethyl group when leading with the parent Cyproflanilide exhibited certain insecticidal activities against *Nilaparvata lugens* (*N. lugens*). The thioethyl derivative (Figure 1) displayed the most potent insecticidal activity (98.92%, 100 mg·L^−1^), as determined through structure–activity relationship (SAR) studies. Regrettably, the activities of the title compound towards *P. xylostella*, *Tetranychus cinnabarinus* (*T. cinnabarinus*) and *A. craccivora* were negligible at the same test concentration, indicating significant room for further exploration in the development of novel *m*-diamide compounds containing sulfur derivatives as potential insecticides.

Due to the diverse biological activities and easily derivable characteristics of sulfide-containing structures, which are often applied in various pesticides, these structures are considered pivotal in the field [6,7]. For example, the first commercialized diamide insecticide, Flubendiamide [8], marking the advent of the era of green diamide insecticides, contained an (O)_2_S-CH_3_ structural unit. Commonly derived sulfur structures such as (O)_n_S-CF_3_ and (O)_n_S-CH_2_CF_3_ have been instrumental in the development of compounds like Fipronil [9], Flupentiofenox [10] and Bisulflufen [11], all of which have demonstrated excellent biological activities in agriculture (Figure 1). 

Considering the significance of thioethers, sulfoxides and sulfones as common sulfides, a series of sulfide-containing *m*-diamide insecticides were designed based on the principle of active fragment transition (Figure 1). Common sulfide active groups in pesticides—(O)_n_S-CH_3_/CF_3_/CH_2_CF_3_—were introduced into the leading with parent—Broflanilide—to replace the CF_3_ group. The exploration of the target synthesis route and insecticidal activities, along with the summarization of SAR, may provide valuable references for future research on sulfur-containing *m*-diamide compounds.

## 2. Results and Discussion

### 2.1. Synthesis

**Scheme 1:**
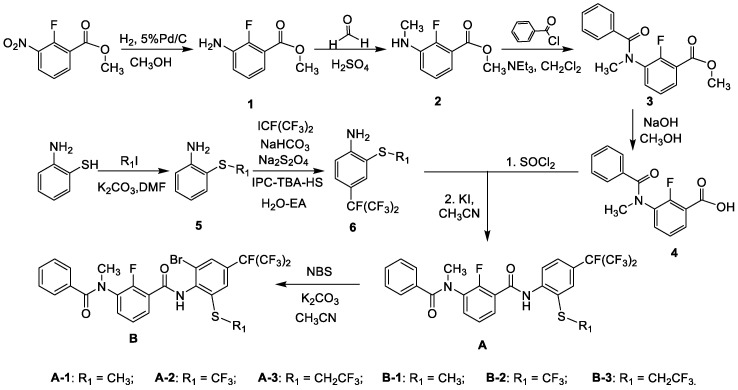
The synthesis route for novel m-diamide compounds containing thioether (**A** and **B**).

The bilinear synthesis routes for the target thioether-containing *m*-diamine compound (**A** and **B**) are shown in Scheme 1 depending on the references and exploration [5,12,13,14]. The key intermediate, 2-fluoro-3-(*N*-methylbenzamido)benzoic acid (**4**), can be smoothly prepared from methyl 2-fluoro-3-nitrobenzoate as the starting material. On the basis of the referenced literature, the synthesis of intermediate **6a** involved extensive explorations (Table 1). The reaction could not proceed when *N*,*N*-Dimethylformamide (DMF) was used as a solvent with **5**, NaOH, FeSO_4_·7H_2_O and (CF_3_)_2_FCI under one-pot reaction conditions [15] (Table 1, entries 1–4). However, employing a two-phase system consisting of ethyl acetate (EA) and water (H_2_O) as solvents, and tetrabutylammonium hydrogen sulfate (IPC-TBA-HS) as a phase transfer catalyst, with the equivalent ratio of compounds **5**:Na_2_S_2_O_4_:NaHCO_3_:IPC-TBA-HS:(CF_3_)_2_FCI being 1:1.1:1.1:0.3:2, the reaction system was successfully completed after heating and refluxing for about 12 h as monitored by thin-layer chromatography (TLC) [5] (Table 1, entries 5–11). The intermediates **6b** and **6c** were prepared following the established synthesis route.

To circumvent the use of harsh conditions, such as the employment of strong bases at low temperatures (e.g., Lithium Diisopropylamide (LDA) or Sodium Hydride (NaH) at −70 °C) [16] or solvents with high boiling points [2], the study aimed to prepare target compounds **A** under milder conditions. A comprehensive review of the literature and an analysis of the reaction mechanism facilitated the exploitation of differences in the nucleophilicity and leaving abilities of halogen atoms for the synthesis of target compounds **A**. The exploration ultimately confirmed that compounds **A** could be efficiently achieved using potassium iodide (KI) [2] as the nucleophilic initiator in an acetonitrile (CH_3_CN) solvent system.

**Scheme 2:**
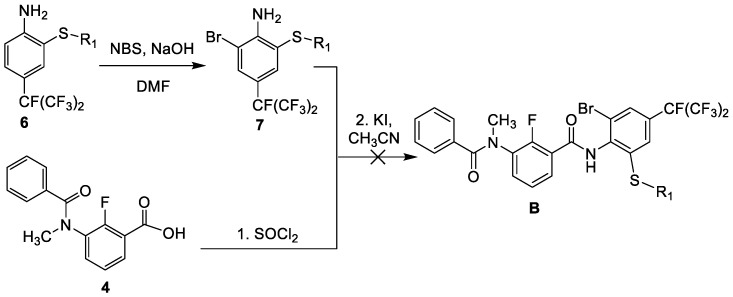
The exploration synthesis route for novel m-diamide compounds containing thioether **B**.

In the endeavor to synthesize target compounds **B**, the research initially attempted to employ the method depicted in Scheme 2. This method started from carboxylic acid **4** with 6-bromo-2-(substitutedthio)-4-(perfluoropropan-2-yl)aniline **7**, utilizing the mild reaction conditions previously successful for compounds **A**. Unfortunately, despite variations in the amount of the nucleophilic reagent (KI), as well as adjustments to the reaction temperature and duration, the reaction did not proceed. The failure was hypothesized to be due to significant steric hindrance around the amino group in the substituted aniline moiety, which hindered the nucleophilic substitution reaction under the mild conditions. Consequently, the focus of the study shifted to using target compounds **A** as the starting material. This approach involved an electrophilic substitution reaction at the six-position of the benzene ring to introduce a bromine atom, leading to the successful preparation of target compounds **B**. Further investigation into the electrophilic substitution reaction on the benzene ring for Br introduction revealed that the target compounds **B** could be synthesized efficiently by using 1.1 equivalents of N-Bromosuccinimide (NBS) and 2.5 equivalents of potassium carbonate (K_2_CO_3_) under reflux conditions for 3 h.

**Scheme 3:**
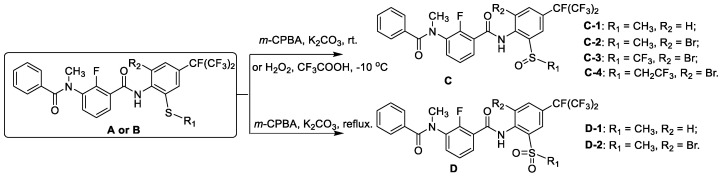
The synthesis route for novel m-diamide compounds containing sulfoxide (**C**) and sulfone (**D**).

As illustrated in Scheme 3, the oxidation reactivity of sulfur atoms is closely related to the nature of the R_1_ group. When R_1_ is an electron-donating methyl, the electron density on the sulfur atom increases, facilitating oxidation. In the presence of 3-chloroperbenzoic acid (m-CPBA) as the oxidant, this leads to the formation of the corresponding sulfoxides (**C-1** and **C-2**, at room temperature) and sulfones (**D-1** and **D-2**, under reflux conditions). However, when R_1_ is an electron-withdrawing trifluoromethyl group, it was observed that oxidation did not occur under m-CPBA conditions (Table 2, entries 1–5). Subsequent explorations confirmed that using **5** equivalents of H_2_O_2_ as the oxidant in trifluoroacetic acid solvent at −10 °C (Table 2, entries 6–13) yielded the sulfoxide (**C-3**) at a low yield of 29%. The sulfoxide compound **C-4**, where R_1_ is CH_2_CF_3_, can be prepared under similar conditions to those used for **C-3** using H_2_O_2_ as the oxidant. Regrettably, the reaction conditions for the oxidation of corresponding compounds from thioethers to corresponding sulfone compounds were not successfully explored when R_1_ was trifluoromethyl or trifluoroethyl. 

The synthesized target compounds (**A**–**D**) underwent comprehensive structural characterization through melting points, ^1^H NMR and ^13^C NMR to elucidate their molecular structures. Meanwhile, the consistency between the theoretical and measured values of HRMS further proved the correctness. In ^1^H NMR spectra, the characteristic proton peak of the amide bond N-H appeared at δ 10.06–10.87 ppm for all m-diamide products. The signals of N-H were observed in δ 10.06–10.63 among compounds **A**. Based on chemical structure analysis, it was speculated that this might be due to the electron-withdrawing properties of Br adjacent to the amino group in compounds **B**, causing the chemical shift to migrate towards a lower field and higher position (δ 10.53–10.71). Notably, the N-CH_3_ signal exhibited a chemical shift of δ 3.32–3.44 ppm, while the range chemical shift for (O)_n_=S-CH_3_ affected by the different oxidation states of sulfur was broad (δ 2.50–3.43). The typical proton peak of (O)_n_=S-CH_2_CF_3_ in **A-3**, **B-3** and **C-4** were presented in δ 4.04–4.37 due to the electron-withdrawing effect of CF_3_.

### 2.2. Structure-Activity Relationship 

The insecticidal activities data of target compounds **A**–**D** against *P. xylostella*, *N. lugens*, and *A. craccivora* are presented in Table 3 and Table 4. All target compounds exhibited 83.33–100.00% larvicidal activity against *P. xylostella* at 500 mg·L*^−^*^1^. And compounds **C-2**, **C-3**, **C-4** and **D-2** revealed particularly excellent inhibition rates, maintaining 60.00–100.00% efficacy even at the concentration of 0.625 mg·L*^−^*^1^. Meanwhile, for *N. lugens* at 500 mg·L*^−^*^1^, all compounds showed poor lethal rates, leading to the conclusion that the sulfide-containing compounds had no significant inhibitory effect on Delphacidae. The target compounds exhibited certain lethal rates (13.79–100.00%) against *A. craccivora* at 500 mg·L*^−^*^1^. It was noteworthy that compounds **B-2**, **C-2**, **C-3** and **D-2** (100%) showed better insecticidal activities than when leading with the parent Broflanilide and comparable effectiveness to Dinotefuran.

As shown in Table 3 and Table 4, the following SAR can be derived. For *P. xylostella*, with the R_1_ group remaining constant, the larvicidal activity sequence of thioether compounds for R_2_ was Br better than H (**B-1** (50 mg·L*^−^*^1^: 30.00%) > **A-1** (50 mg·L*^−^*^1^: 0.00%); **B-2** (50 mg·L*^−^*^1^: 100.00%) > **A-2** (200 mg·L*^−^*^1^: 86.67%); **B-3** (500 mg·L*^−^*^1^: 100.00%) > **A-3** (500 mg·L*^−^*^1^: 83.33%)). The pattern was also observed in sulfoxides and sulfone products: **C-2** (0.625 mg·L*^−^*^1^: 60.00%) > **C-1** (50 mg·L*^−^*^1^: 20.00%); **D-2** (0.625 mg·L*^−^*^1^: 73.33%) > **D-1** (200 mg·L*^−^*^1^: 56.67%). The structural analysis of compounds **B-1**, **C-2** and **D-2**, where R_1_ = CH_3_ and R_2_ = Br, indicated that better insecticidal activity corresponds with higher oxidation states of sulfur: sulfone compound (**D-2** (0.625 mg·L*^−^*^1^: 73.33%)) > sulfoxide compound (**C-2** (0.625 mg·L*^−^*^1^: 60.00%)) > thioether compound (**B-1** (50 mg·L*^−^*^1^: 30.00%)). SAR analysis revealed that among all compounds, the sulfoxide compounds **C-2**, **C-3** and **C-4** exhibited particularly excellent insecticidal activity against P. xylostella. Even at a concentration of 0.625 mg·L*^−^*^1^, they still exhibited wonderful insecticidal activity of 60–100.00%. When R_1_ = CH_2_CF_3_, it was found that the lethal rate against *P. xylostella* sulfoxide compound **C-4** was significantly superior to that of thioether compounds (**A-3** and **B-3**). It was speculated that this might be because the stronger electron-withdrawing capacity of sulfoxide was favorable for the improvement in the bioactivity. For R_2_ = Br, the sulfoxide compounds **C** with R_1_ = CF_3_ (100.00%) or CH_2_CF_3_ (100.00%) were more effective than those with R_1_ = CH_3_ (60.00%). This indicated that the insecticidal activities against *A. craccivora* were significantly better for **B-2**, **C-2** and **D-2**, as shown in Table 4, particularly for those with R_1_ = CH_3_ and R_2_ = Br. In particular, the sulfoxide compound **C-3** with a stronger electron-withdrawing R_1_ = CF_3_ is favorable for the improvement in the bioactivity.

Based on the above, it could be concluded that some sulfide-containing *m*-diamine compounds as designed in the study contained excellent insecticidal activities against *P. xylostella* and *A. craccivora*. Furthermore, the sulfoxide compounds **C-2**, **C-3** and **C-4**, along with the sulfone compound **D-2**, demonstrated significantly higher bioactivities compared to the others.

## 3. Materials and Methods

### 3.1. General Experimental Details

Reagents and solvents were purchased from Titan Corporation and used without further purification. Melting points were measured by the SGWX-4B melting point analyzer and uncorrected. NMR spectra were recorded on a Brucker Avance NEO (400, 101 MHz) spectrometer, using DMSO-*d6* (TMS as the 0 point internal standard) as the solvent. HRMS data were obtained on Thermo Q Exactive Focus with ESI ionization. The ^1^H NMR, ^13^C NMR and HRMS spectra for target compounds were provided in Supplementary Materials.

### 3.2. Synthesis and Characterization of the Compounds

A mass of synthesis routes for *m*-diamide pesticides have been reported in recent years due to their exceptional insecticidal activities [5,12,13,14]. The sulfur-containing *m*-diamide target compounds were designed based on literature reports and exploration. The use of methyl 2-fluoro-3-nitrobenzoate as the starting material to obtain the target compounds through a bilinear chain reaction is depicted in Scheme 1 and Scheme 2.

#### 3.2.1. General Procedure for the Preparation of Thioether-Containing *m*-Diamine Compound **A** and **B**

Freshly prepared benzoyl chloride was obtained by dissolving 2-fluoro-3-(*N*-methylbenzamido)benzoic acid (**4**, 20 mmol) in 15 mL of SOCl_2_ and refluxing for 6 h. This mixture was then transferred to a 100 mL round-bottom flask containing 2-(substitutedthio)-4-(perfluoropropan-2-yl)aniline (**6**, 20 mmol), KI (30 mmol) and 50 mL of CH_3_CN [5,12]. After that, the reaction mixture was heated to reflux and maintained for about 6 h, monitored by TLC. Then, it was concentrated under reduced pressure. The residue was dissolved in 30 mL of CH_2_Cl_2_ and washed with brine. The organic phase was dried with Na_2_SO_4_ and subjected to column chromatography to obtain the target products **A**.

*2-fluoro-3-(N-methylbenzamido)-N-(2-(methylthio)-4-(perfluoropropan-2-yl)phenyl)benzamide* (**A-1**). Yellow solid (58%), m.p. 103–104 °C. ^1^H NMR (400 MHz, DMSO) *δ* 10.06 (s, 1H, -NH), 7.83 (d, *J* = 7.7 Hz, 1H, Ph-H), 7.67–7.61 (m, 1H, Ph-H), 7.60–7.55 (m, 2H, Ph-H), 7.53 (s, 1H, Ph-H), 7.42–7.22 (m, 6H, Ph-H), 3.35 (s, 3H, CH_3_), 2.50 (s, 3H, CH_3_). ^13^C NMR (101 MHz, DMSO) *δ* 170.6, 162.5, 138.4, 138.3, 136.1, 135.9, 133.4, 130.4, 129.8, 128.5, 128.1, 127.0, 125.4 (d, *J* = 4.3 Hz), 124.6, 124.2 (q, *J* = 272.3 Hz, CF_3_), 123.6, 123.4, 123.2, 122.0, 119.1, 39.3, 15.8. HRMS calcd for C_25_H_18_F_8_N_2_O_2_S [M + H]^+^ 563.1039, found 563.1035.

*2-fluoro-3-(N-methylbenzamido)-N-(4-(perfluoropropan-2-yl)-2-((trifluoromethyl)thio)phenyl) benzamide* (**A-2**). Yellow oil (66%). ^1^H NMR (400 MHz, DMSO) *δ* 10.63 (s, 1H, -NH), 8.07 (d, *J* = 8.5 Hz, 1H, Ph-H), 8.03–7.98 (m, 2H, Ph-H), 7.68–7.60 (m, 2H, Ph-H), 7.35–7.25 (m, 6H, Ph-H), 3.36 (s, 3H, CH_3_). ^13^C NMR (101 MHz, DMSO) *δ* 170.6, 162.7, 153.6, 143.9, 135.9, 134.1, 134.0, 133.8, 131.1, 130.4, 130.3, 130.2, 129.8, 128.4, 128.0, 126.6, 125.6, 125.5, 123.9, 123.0, 120.1, 39.6. HRMS calcd for C_25_H_15_F_11_N_2_O_2_S [M + H]^+^ 617.0757, found 617.0750.

*2-fluoro-3-(N-methylbenzamido)-N-(4-(perfluoropropan-2-yl)-2-((2,2,2-trifluoroethyl)thio)phen--yl)benzamide* (**A-3**). Yellow solid (58%), m.p. 119–120 °C. ^1^H NMR (400 MHz, DMSO) *δ* 10.24 (s, 1H, -NH), 8.03 (d, *J* = 8.6 Hz, 1H, Ph-H), 7.90 (d, *J* = 1.6 Hz, 1H, Ph-H), 7.72–7.66 (m, 2H, Ph-H), 7.61 (t, *J* = 5.9 Hz, 1H, Ph-H), 7.40–7.30 (m, 3H, Ph-H), 7.29–7.25 (m, 3H, Ph-H), 4.1 (q, *J* = 9.3 Hz, 2H, -CH_2_CF_3_), 3.35 (s, 3H, CH_3_). ^13^C NMR (101 MHz, DMSO) *δ* 170.6, 162.4, 153.7, 141.2, 135.9, 133.7, 133.3, 130.4, 130.3, 129.9, 128.5, 128.0, 127.7, 126.5, 126.0, 125.5, 125.4, 124.2, 122.9, 122.7, 122.0, 39.34, 35.6 (q, CH_2_, *J* = 31.3 Hz). HRMS calcd for C_26_H_17_F_11_N_2_O_2_S [M + H]^+^ 631.0913, found 631.0907.

The thioether-containing *m*-diamide compound (**A**, 10 mmol) was dissolved in 30 mL of CH_3_CN and then cooled down to 0 °C. K_2_CO_3_ (25 mmol) and NBS (11 mmol) were added slowly in batches. The reaction system was then warmed to reflux stirring for 4 h (monitored by TLC) [12]. After completion, the mixture was concentrated and the residue was dissolved in 20 mL of CH_2_Cl_2_. The organic phase was washed with brine and dried by Na_2_SO_4_. The thioether-containing compounds **B** were obtained by further purification through column chromatography.

*N-(2-bromo-6-(methylthio)-4-(perfluoropropan-2-yl)phenyl)-2-fluoro-3-(N-methylbenzamido) benzamide* (**B-1**). Yellow solid (70%), m.p. 167–169 °C. ^1^H NMR (400 MHz, DMSO) *δ* 10.64 (s, 1H, -NH), 7.85 (d, *J* = 7.7 Hz, 1H, Ph-H), 7.66–7.60 (m, 3H, Ph-H), 7.54–7.51 (m, 1H, Ph-H), 7.30–7.24 (m, 5H, Ph-H), 3.35 (s, 3H, CH_3_), 2.79 (s, 3H, CH_3_). ^13^C NMR (101 MHz, DMSO) *δ* 170.7, 163.2, 140.4, 139.1, 135. 9, 134.6, 134.5, 133.2, 130.4, 129.2, 129.1, 128.4, 127.9, 126.9, 125.4, 125.3, 124.4, 123.2, 119.7, 113.4, 39.2, 14.24. HRMS calcd for C_25_H_17_BrF_8_N_2_O_2_S [M + H]^+^ 641.0145, found 641.0140.

*N-(2-bromo-4-(perfluoropropan-2-yl)-6-((trifluoromethyl)thio)phenyl)-2-fluoro-3-(N-methylben--zamido)benzamide* (**B-2**). Yellow solid (82%), m.p. 85–86 °C. ^1^H NMR (400 MHz, DMSO) *δ* 10.71 (s, 1H, -NH), 8.29–8.06 (m, 2H, Ph-H), 7.85 (s, 1H, Ph-H), 7.61–7.58 (m, 2H, Ph-H), 7.48–7.40 (m, 5H, Ph-H), 3.33 (s, 3H, CH_3_). ^13^C NMR (101 MHz, DMSO) *δ* 170.6, 163.5, 155.9, 153.4, 138.9, 135.9, 133.3, 132.9, 131.8, 131.6, 130.5, 129.7, 129.4, 129.0, 128.5, 128.0, 126.8, 125.3, 124.9, 123.5, 122.3, 39.5. HRMS calcd for C_25_H_14_BrF_11_N_2_O_2_S [M + H]^+^ 694.9862, found 694.9854.

*N-(2-bromo-4-(perfluoropropan-2-yl)-6-((2,2,2-trifluoroethyl)thio)phenyl)-2-fluoro-3-(N-methyl--benzamido)benzamide* (**B-3**). Yellow solid (70%), m.p. 142–144 °C. ^1^H NMR (400 MHz, DMSO) *δ* 10.53 (s, 1H, NH), 7.86 (s, 1H, Ph-H), 7.79 (s, 1H, Ph-H), 7.63–7.58 (m, 2H, Ph-H), 7.41–7.23 (m, 6H, Ph-H), 4.30–4.10 (m, 2H, -CH_2_CF_3_), 3.36 (s, 3H, CH_3_). ^13^C NMR (101 MHz, DMSO) *δ* 170.7, 162.6, 156.0, 153.5, 139.1, 138.1, 135.9, 133.2, 130.4, 129.2, 128.5, 128.0, 127.7, 126.5, 126.3, 125.6, 125.4, 124.9, 124.6, 124.4, 119.1, 39.5, 33.5 (q, CH_2_, *J* = 31.3 Hz). HRMS calcd for C_26_H_17_BrF_11_N_2_O_2_S [M + H]^+^ 709.0014, found 709.0018.

#### 3.2.2. General Procedure for the Preparation of Sulfoxide-Containing *m*-Diamine Compound **C**

The thioether-containing *m*-diamide compound (**A-1**, 1 mmol) was dissolved in 10 mL of 1,4-dioxane with 3 mmol *m*-CPBA and 1 mmol K_2_CO_3_ and maintained at room temperature for about 3 h (monitored by TLC). After that, it was concentrated, and the residue was dissolved 20 mL of CH_2_Cl_2_ [5,13]. The organic phase was then washed with brine and dried by Na_2_SO_4_. The sulfoxide-containing compound **C-1** was obtained by further purification through column chromatography. The same went for the synthesis method of compound **C-2**, starting with **B-1** as the raw material.

*2-fluoro-3-(N-methylbenzamido)-N-(2-(methylsulfinyl)-4-(perfluoropropan-2-yl)phenyl)benzam-ide* (**C-1**). White solid (62%), m.p. 112–113 °C. ^1^H NMR (400 MHz, DMSO) *δ* 10.87 (s, 1H, -NH), 8.08 (s, 1H, Ph-H), 7.95–7.90 (m, 2H, Ph-H), 7.63–7.58 (m, 2H, Ph-H), 7.38–7.24 (m, 6H, Ph-H), 3.35 (s, 3H, CH_3_), 2.83 (s, 3H, CH_3_). ^13^C NMR (101 MHz, DMSO) *δ* 170.6, 163.2, 155.9, 153.4, 141.8, 138.1, 135.9, 133.6, 130.4, 129.6, 128.5, 128.0, 127.5, 125.5, 124.3, 124.2, 124.1, 123.9, 122.4, 119.3, 42.3, 39.3. HRMS calcd for C_25_H_18_F_8_N_2_O_3_S [M + H]^+^ 579.0989, found 579.0986.

*N-(2-bromo-6-(methylsulfinyl)-4-(perfluoropropan-2-yl)phenyl)-2-fluoro-3-(N-methylbenzamido) benzamide* (**C-2**). White solid (74%), m.p. 105–107 °C. ^1^H NMR (400 MHz, DMSO) *δ* 10.61 (s, 1H, -NH), 8.24 (s, 1H, Ph-H), 8.05 (s, 1H, Ph-H), 7.66 (t, *J* = 6.7 Hz, 1H, Ph-H), 7.60 (t, *J* = 6.3 Hz, 1H, Ph-H), 7.45–7.29 (m, 6H, Ph-H), 3.37 (s, 3H, CH_3_), 2.77 (s, 3H, CH_3_). ^13^C NMR (101 MHz, DMSO) *δ* 170.7, 163.2, 155.9, 149.9, 136.3, 135.9, 133.4, 133.2, 133.1, 132.4, 130.4, 129.2, 128.5, 128.0, 127.6, 126.1, 125.6, 125.5, 120.7, 119.1, 42.9, 39.3. HRMS calcd for C_25_H_17_BrF_8_N_2_O_3_S [M + H]^+^ 657.0094, found 657.0086.

Next, 0.30 mmol of **B-2** was dissolved in 10 mL of CF_3_COOH and cooled down to −10 °C. Then, 1.50 mmol of 30% H_2_O_2_ was added slowly. The reaction was conducted at −10 °C for 3 h [14], with the progress being monitored using TLC. Upon completion, the mixture was concentrated under reduced pressure. The sulfoxide-containing compounds **C-3** were obtained by further purification through column chromatography. The same went for the synthesis method of compound **C-4**, starting with **B-3** as the raw material.

*N-(2-bromo-4-(perfluoropropan-2-yl)-6-((trifluoromethyl)sulfinyl)phenyl)-2-fluoro-3-(N-methyl--benzamido)benzamide* (**C-3**). Yellow solid(29%), m.p. 103–105 °C. ^1^H NMR (400 MHz, DMSO) *δ* 8.27 (s, 1H, Ph-H), 7.96 (s, 1H, Ph-H), 7.59–7.54 (m, 2H, Ph-H), 7.35–7.26 (m, 6H, Ph-H), 3.35 (s, 3H). ^13^C NMR (101 MHz, DMSO) *δ* 170.6, 163.0, 156.1, 152.8, 136.0, 134.8, 133.6, 132.4, 130.8, 130.3, 129.6, 129.5, 129.3, 128.4, 127.9, 125.1, 123.1, 122.2, 122.0, 119.2, 112.9, 39.3. HRMS calcd for C_25_H_14_BrF_11_N_2_O_3_S [M + H]^+^ 712.9795, found 712.9765.

*N-(2-bromo-4-(perfluoropropan-2-yl)-6-((2,2,2-trifluoroethyl)sulfinyl)phenyl)-2-fluoro-3-(N-methylbenzamido)benzamide* (**C-4**). Yellow solid (30%), m.p. 80–82 °C. ^1^H NMR (400 MHz, DMSO) *δ* 10.66 (s, 1H, NH), 8.34 (s, 1H, Ph-H), 8.14 (s, 1H, Ph-H), 7.91 (s, 1H, Ph-H), 7.80–7.70 (m, 1H, Ph-H), 7.58–7.51 (m, 1H, Ph-H), 7.40–7.32 (m, 5H, Ph-H), 4.37 (dd, *J* = 22.5, 9.0 Hz, 1H, -CH_2_CF_3_), 4.04 (dd, *J* = 22.5, 9.0 Hz, 1H, -CH_2_CF_3_), 3.44 (s, 3H, CH_3_). ^13^C NMR (101 MHz, DMSO) *δ* 170.6, 166.6, 163.3, 153.6, 145.7, 136.4, 135.8, 133.8, 133.4, 133.2, 131.1, 130.4, 129.3, 128.4, 127.9, 127.8, 127.6, 126.3, 125.6, 123.2, 121.9, 57.5 (q, *J* = 26.2 Hz, -CH_2_CF_3_), 39.3. HRMS calcd for C_26_H_17_BrF_11_N_2_O_3_S [M + H]^+^ 724.9968, found 724.9963.

#### 3.2.3. General Procedure for the Preparation of Sulfone-Containing *m*-Diamine Compound **D**

The thioether-containing *m*-diamide compound **A-1** (1 mmol) was dissolved in 10 mL of 1,4-dioxane with 5 mmol of *m*-CPBA and 1 mmol of K_2_CO_3_ warmed to reflux and maintained for about 5 h (monitored by TLC). After that, it was concentrated, and the residue was dissolved 20 mL of CH_2_Cl_2_ [5,13]. The organic phase was washed with brine and dried by Na_2_SO_4_. The sulfoxide-containing compound **D-1** was afforded by further purification through column chromatography. The same went for the synthesis method of compound **D-2**, starting with **B-1** as the raw material.

*2-fluoro-3-(N-methylbenzamido)-N-(2-(methylsulfonyl)-4-(perfluoropropan-2-yl)phenyl)benza-mide* (**D-1**). Yellow solid(60%), m.p. 87–89 °C. ^1^H NMR (400 MHz, DMSO) *δ* 10.40 (s, 1H, -NH), 8.61 (d, *J* = 9.4 Hz, 1H, Ph-H), 8.12 (s, 1H, Ph-H), 7.91 (s, 1H, Ph-H), 7.90–7.88 (m, 1H, Ph-H), 7.72–7.69 (m, 2H, Ph-H), 7.55–7.52 (m, 1H, Ph-H), 7.34–7.27 (m, 4H, Ph-H), 3.43 (s, 3H, CH_3_), 3.35 (s, 3H, CH_3_). ^13^C NMR (101 MHz, DMSO) *δ* 170.6, 166.6, 161.9, 139.7, 135.8, 134.5, 133.8, 133.4, 133.2, 132.6, 131.1, 130.3, 129.3, 128.5, 128.4, 127.9, 127.2, 126.0, 123.3, 121.7, 43.7, 39.3. HRMS calcd for C_25_H_18_F_8_N_2_O_4_S [M + H]^+^ 595.0938, found 595.0930.

*N-(2-bromo-6-(methylsulfonyl)-4-(perfluoropropan-2-yl)phenyl)-2-fluoro-3-(N-methylbenzamid--o)benzamide* (**D-2**). White solid (58%), m.p. 207–209 °C. ^1^H NMR (400 MHz, DMSO) *δ* 10.70 (s, 1H, -NH), 8.46 (d, *J* = 1.9 Hz, 1H, Ph-H), 8.20 (d, *J* = 1.9 Hz, 1H, Ph-H), 7.66–7.63 (s, 2H, Ph-H), 7.40–7.28 (s, 6H, Ph-H), 3.38 (s, 3H, CH_3_), 3.37 (s, 3H, CH_3_). ^13^C NMR (101 MHz, DMSO) *δ* 170.8, 163.3, 155.9, 149.9, 136.4, 136.0, 133.5, 133.4, 132.5, 130.5, 129.3, 128.6, 128.1, 127.7, 126.2, 125.8, 125.7, 120.8, 120.7, 119.2, 43.1, 39.4. HRMS calcd for C_25_H_17_BrF_8_N_2_O_4_S [M + H]^+^ 673.0043, found 673.0036.

### 3.3. Insecticidal Activity Assay [17,18,19]

#### 3.3.1. Rearing Conditions

*P. xylostella*: Raised indoors with radish seedlings at a temperature of 22 ± 2 °C and a light intensity of 12L:12D.

*N. lugens*: Raised indoors with water rice seedlings at a temperature of 26 ± 2 °C and a light intensity of 12L:12D.

*A. craccivora*: Reared indoors with silkworm bean seedlings at a temperature of 22 ± 2 °C and a light intensity of 12L:12D.

#### 3.3.2. Drug Preparation

The raw materials were dissolved in DMF to prepare a 1% mother liquor, diluted with 0.1% Tween 80 distilled water to prepare the corresponding concentration and set aside.

#### 3.3.3. Insecticidal Activity Methods

The lethal rate of the target compounds against *P. xylostella*, *N. lugens* and *A. craccivora* were investigated under the contrast of Broflanilide and a blank control without any medication in a greenhouse.

The activity of *P. xylostella* was determined using the leaf-soaking method. Radish leaves were soaked in moderation to the prepared test fluid for 30 s. Then, they were placed in a plastic culture dish with filter paper and dried naturally in the shade. Each dish was infested with 8 second-instar diamondback moth larvae and placed in an observation room at a temperature of 25 °C. The test results were observed after 48 h. They were considered dead if there was no response or the inability to crawl normally was observed when touching the insect body lightly with a brush. This was repeated three times for each sample.

The activity of *N. lugens* was determined using the spray method. A rice seedling with two leaves and one core was selected and placed on a 6 cm Petri dish; then, quartz sand was spread on the Petri dish. Each dish was infested with 20 third-instar early brown planthopper nymphs and treated with 2.5 mL of spray with a Potter spray tower, which were then placed in an observation room at a temperature of 25 °C. The test results were observed after 48 h. They were considered dead if there is no response or the inability to crawl normally was observed when touching the insect body lightly with a brush. This was repeated three times for each sample.

The activity of *A. craccivora* was determined using the spray method. The dishes were infested with 30 alfalfa aphid nymphs and treated with 2.5 mL of spray with a Potter spray tower and then placed in a 25 °C observation room for cultivation. After 48 h of investigation, the insect body was touched with tweezers, and if there was no response, it was regarded as dead. A blank control was set up without adding any medication.

#### 3.3.4. Data Statistics and Analysis

The number of deaths of each processed target was counted, and the lethal rate was calculated.

$$\mathrm{Lethal}\;\mathrm{rate}\;(\%)=\frac{number\;of\;dead\;insects}{total\;number\;of\;insects}\times 100\%$$

## 4. Conclusions

A series of novel sulfide-containing *m*-diamine target compounds **A**–**D** were prepared through exploration by referencing and improving upon the literature. These compounds were characterized by melting points, ^1^H NMR, ^13^C NMR and HRMS. The mild preparation of the thioether compounds **A** was achieved by cleverly utilizing I^-^ for its excellent nucleophilicity and leaving properties, thereby avoiding the need for harsh conditions such as strong bases and low temperatures. The insecticidal activity results revealed that most target compounds **A**–**D** exhibited 100% lethal rates against *P. xylostella* and *A. craccivora* at 500 mg·L^−1^. Specifically, for *P. xylostella*, the sulfoxide-compounds **C-2**, **C-3** and **C-4** and sulfone compound **D-2** displayed insecticidal activities ranging from 60.00% to 100.00% even at a concentration of 0.625 mg·L^−1^. It was observed that compounds with R_1_ = CH_3_ and R_2_ = Br (**B-1**, **C-2** and **D-2**) and sulfoxide compound **C-3** achieved 100.00% lethal rates against *A. craccivora* at 500 mg·L^−1^, outperforming the lethal rates achieved when leading with the parent Broflanilide as per the SAR. Some newly synthesized sulfide-containing *m*-diamine target compounds had a broader insecticidal spectrum. From this, it can be inferred that the novel sulfoxide compounds **C-2**, **C-3** and **D-2** are promising candidates for further study as potential active molecules in new insecticides.

## Figures and Tables

**Table 1:** Optimization of reaction conditions for the synthesis compound **6a**.

Entry	Solvent	Reaction Condition (Equiv. of Reactant)	Temp. (°C)	Yield of 6a (%) ^a^
1	DMF	5:NaOH:FeSO_4_·7H_2_O:(CF_3_)_2_FCI 1:8.5:1.5:1.5	rt	nr
2			reflux	nr
3		5:NaOH:FeSO_4_·7H_2_O:(CF_3_)_2_FCI 1:9.5:1.5:1.5	reflux	nr
4		5:NaOH:FeSO_4_·7H_2_O:(CF_3_)_2_FCI 1:9.5:3:3	reflux	nr
5	EA-H_2_O	5:Na_2_S_2_O_4_:NaHCO_3_:IPC-TBA-HS:(CF_3_)_2_FCI 1:1.1:1.1:0.1:1.1	rt	ur
6			40	ur
7			reflux	ur
8		5:Na_2_S_2_O_4_:NaHCO_3_:IPC-TBA-HS:(CF_3_)_2_FCI 1:1.1:1.1:0.3:1.1	reflux	ur
9		5:Na_2_S_2_O_4_:NaHCO_3_:IPC-TBA-HS:(CF_3_)_2_FCI 1:1.1:1.1:0.3:2	reflux	42%
10				
11				

^a^ isolated yields; nr: non-reaction; ur: under-reaction.

**Table 2:** Optimization of reaction conditions for the synthesis compound **C-3**.

Entry	Solvent	Reaction Condition (Equiv. of Reactant)	Temp. (°C)	Yield of C-3 (%) ^a^
1	1,4-dioxane	**B-2**:m-CPBA:K_2_CO_3_ 1:3:1	−10	nr
2			rt	nr
3			reflux	nr
4		**B-2**:m-CPBA:K_2_CO_3_ 1:4:1	reflux	nr
5		**B-2**:m-CPBA:K_2_CO_3_ 1:5:1	reflux	nr
6	CF_3_COOH	**B-2**:H_2_O_2_ 1:1	0	nr
7			rt	ur
8			reflux	ur
		**B-2**:H_2_O_2_ 1:3	−10	dm
9			0	dm
10			rt	dm
11			reflux	dm
12		**B-2**:H_2_O_2_ 1:5	−10	29%
13			rt	dm

^a^ isolated yields; nr: non-reaction; ur: under-reaction; dm: deterioration of raw material.

**Table 3:** Insecticidal activity of target compounds against *P. xylostella*.

No.	Lethal Rate (%) at a Concentration (mg·L^−1^) of *P. xylostella*							
	500	200	50	25	12.5	10	2.5	0.625
**A-1**	100.00	100.00	0.00	-	-	-	-	-
**A-2**	100.00	86.67	-	-	-	-	-	-
**A-3**	83.33	-	-	-	-	-	-	-
**B-1**	100.00	100.00	30.00	-	-	-	-	-
**B-2**	10.00	100.00	100.00	0.00	-	-	-	-
**B-3**	100.00	0.00	-	-	-	-	-	-
**C-1**	100.00	100.00	20.00	-	-	-	-	-
**C-2**	100.00	100.00	100.00	100.00	100.00	100.00	100.00	60.00
**C-3**	100.00	100.00	100.00	100.00	100.00	100.00	100.00	100.00
**C-4**	100.00	100.00	100.00	100.00	100.00	100.00	90.00	100.00
**D-1**	100.00	56.67	-	-	-	-	-	-
**D-2**	100.00	100.00	100.00	100.00	100.00	100.00	100.00	73.33
Broflanilide	100.00	100.00	100.00	100.00	100.00	100.00	100.00	100.00
Dinotefuran	-	-	-	-	-	-	-	-
Blank control	0.00	0.00	0.00	0.00	0.00	0.00	0.00	0.00

-: not test.

**Table 4:** Insecticidal activity of target compounds against N. lugens and A. craccivora.

No.	Lethal Rate (%) at a Concentration of (mg·L^−1^)				
	*A. craccivora*				*N. lugens*
	500	200	50	12.5	500
**A-1**	36.36	-	-	-	4.76
**A-2**	31.18	-	-	-	0.00
**A-3**	13.79	-	-	-	0.00
**B-1**	-	-	-	-	0.00
**B-2**	100.00	29.11	29.47	13.10	4.55
**B-3**	-	-	-	-	-
**C-1**	12.50	-	-	-	0.00
**C-2**	100.00	15.48	18.57	10.53	0.00
**C-3**	100.00	75.00	59.09	9.09	0.00
**C-4**	33.90	-	-	-	0.00
**D-1**	0.00	-	-	-	0.00
**D-2**	100.00	66.67	48.61	31.1	0.00
Broflanilide	50.00	-	-	-	0.00
Dinotefuran	100.00	100.00	73.53	48.94	100.00
Blank control	0.00	0.00	0.00	0.00	0

-: not test.

## Data Availability

Data are contained within the article and Supplementary Materials.

## Supplementary Materials

The following supporting information can be downloaded at: https://www.mdpi.com/article/10.3390/molecules29061337/s1, the ^1^H NMR, ^13^C NMR and HRMS spectra for target compounds.
