# Supplementary material for: Novel Meta-Diamide Compounds Containing Sulfide Derivatives Were Designed and Synthesized as Potential Pesticides
Source: Molecules. 2024 Mar 17;29(6):1337. doi: 10.3390/molecules29061337 (PMC10974209; doi:10.3390/molecules29061337)
Supplement: Supplementary file 1 [file molecules-29-01337-s001.zip › molecules-2891036-supplementary.pdf]

Molecules

MATERIAL SUPLEMENTAR
For

Novel Meta-Diamide Compounds Containing Sulfide Derivatives Were Designed and Synthesized as Potential Pesticides

Jingwen Wu [1], Shuaihui Dang [1], Yan Zhang [1,*] and Sha Zhou [2]

[1] College of Resources and Environmental Engineering, Shanghai Polytechnic University, Shanghai 201209, China; 20221516055@stu.sspu.edu.cn (J.W.); 20221516008@stu.sspu.edu.cn (S.D.)
[2] Collaborative Innovation Center of Zhejiang Green Pesticide, National Joint Local Engineering Laboratory for High-Efficient Preparation of Biopesticide, School of Forestry and Biotechnology, Zhejiang A & F University, Hangzhou 311300, China; 15822308335@163.com
* Correspondence: yzhang@sspu.edu.cn; Tel.: +86-15902144815

^{1}H NMR, ^{13}C NMR and HRMS spectra for target compounds

Table of contents

1. ^{1}H NMR, ^{13}C NMR and HRMS spectra for compound A-1……………………………….....2

2. ^{1}H NMR, ^{13}C NMR and HRMS spectra for compound A-2……………………………….....3

3. ^{1}H NMR, ^{13}C NMR and HRMS spectra for compound A-3………………………………....5

4. ^{1}H NMR, ^{13}C NMR and HRMS spectra for compound B-1………………………………....6

5. ^{1}H NMR, ^{13}C NMR and HRMS spectra for compound B-2………………………………....8

6. ^{1}H NMR, ^{13}C NMR and HRMS spectra for compound B-3……………………………….....9

7. ^{1}H NMR, ^{13}C NMR and HRMS spectra for compound B-4……………………………….....11

8. ^{1}H NMR, ^{13}C NMR and HRMS spectra for compound C-1………………………………....12

9. ^{1}H NMR, ^{13}C NMR and HRMS spectra for compound C-2………………………………....14

10. ^{1}H NMR, ^{13}C NMR and HRMS spectra for compound C-3………………………………....15

11. ^{1}H NMR, ^{13}C NMR and HRMS spectra for compound D-1………………………………....17

12. ^{1}H NMR, ^{13}C NMR and HRMS spectra for compound D-2………………………………....18

A-1

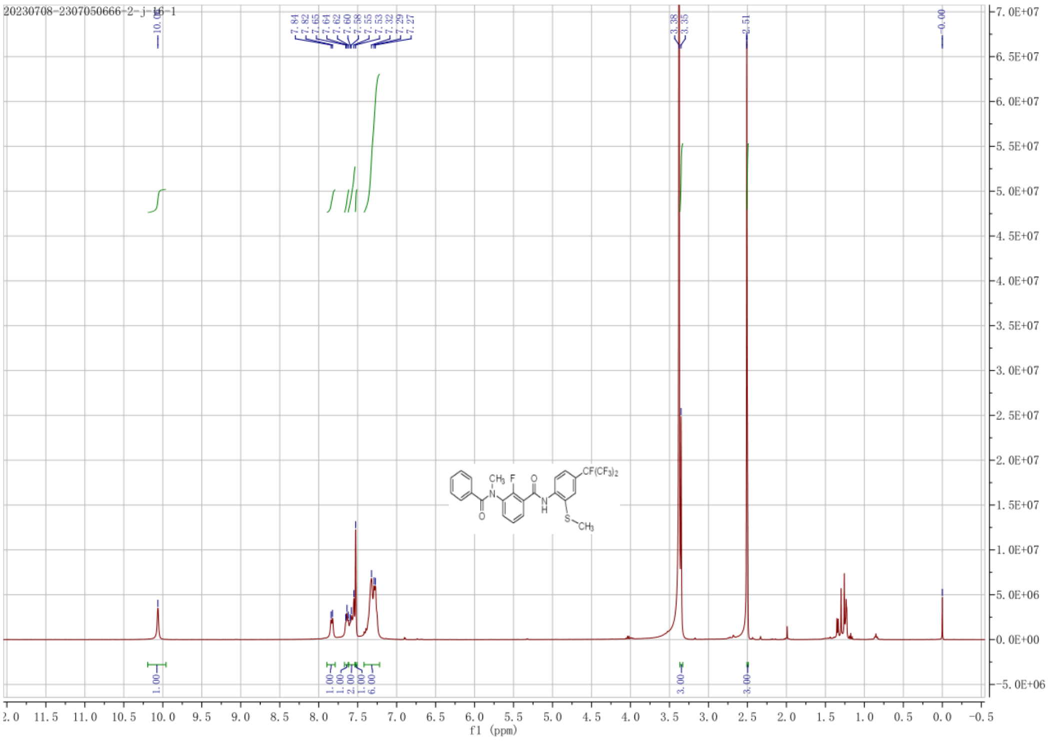

Figure 1S** ^{1}H NMR spectrum of **A-1

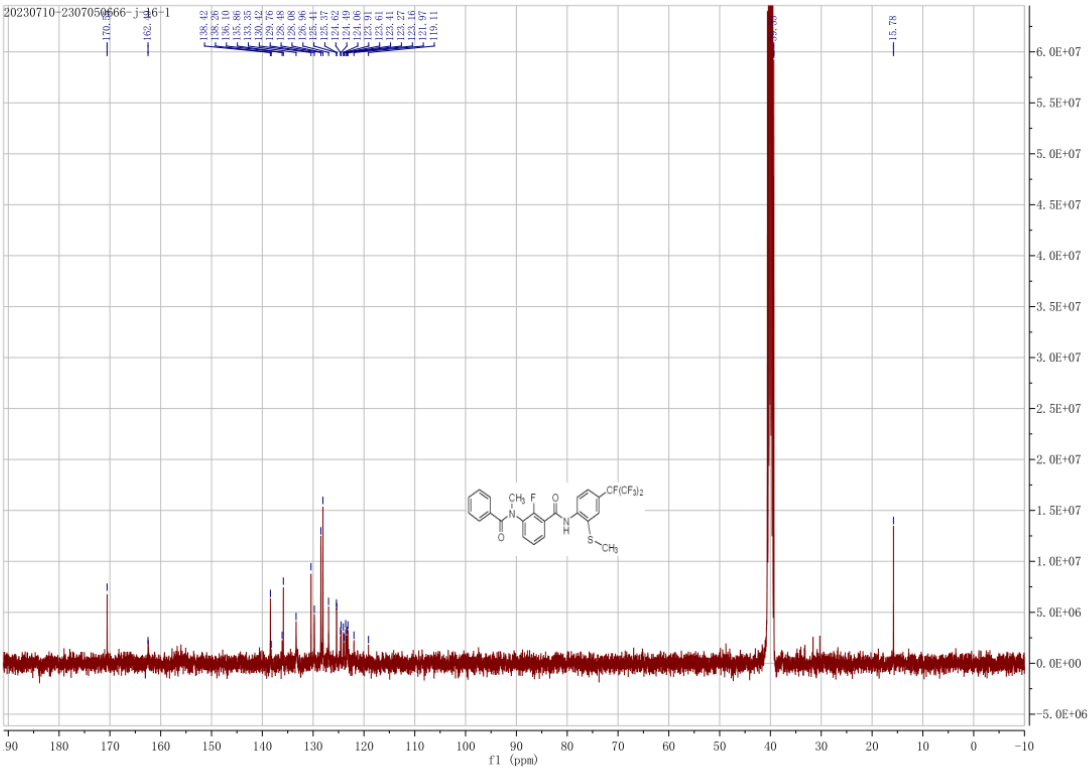

Figure 2S** ^{13}C NMR spectrum of **A-1

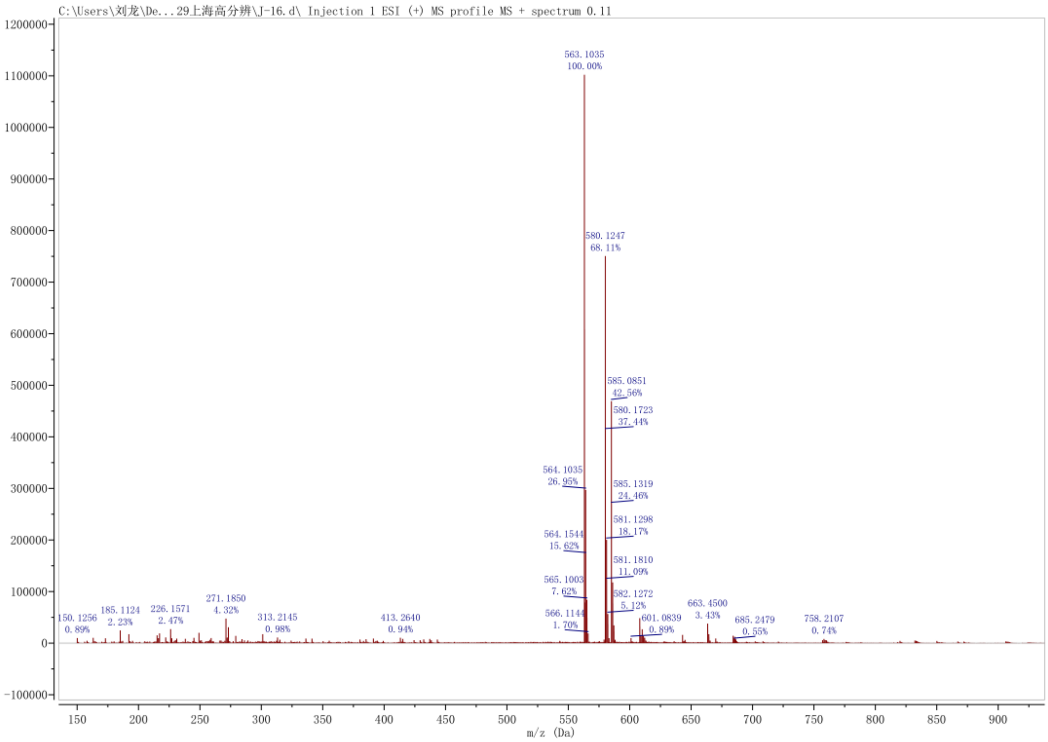

Figure 3S** HRMS spectrum of **A-1

A-2

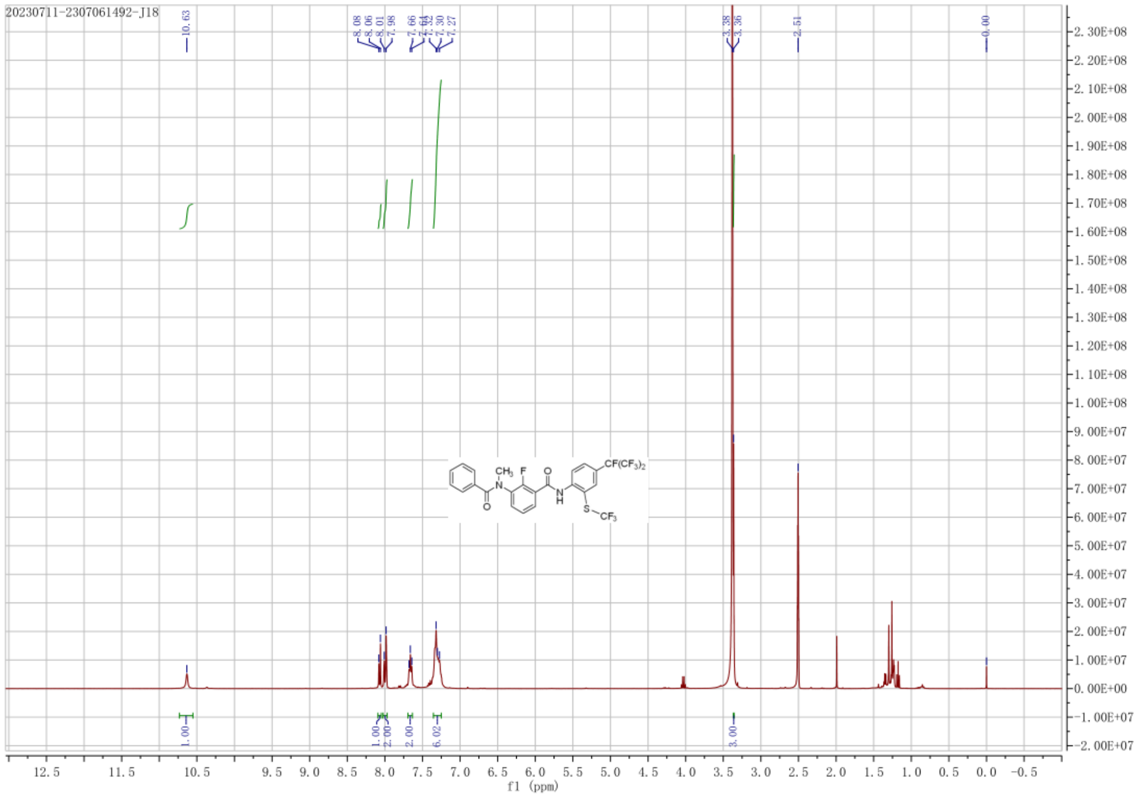

Figure 4S** ^{1}H NMR spectrum of **A-2

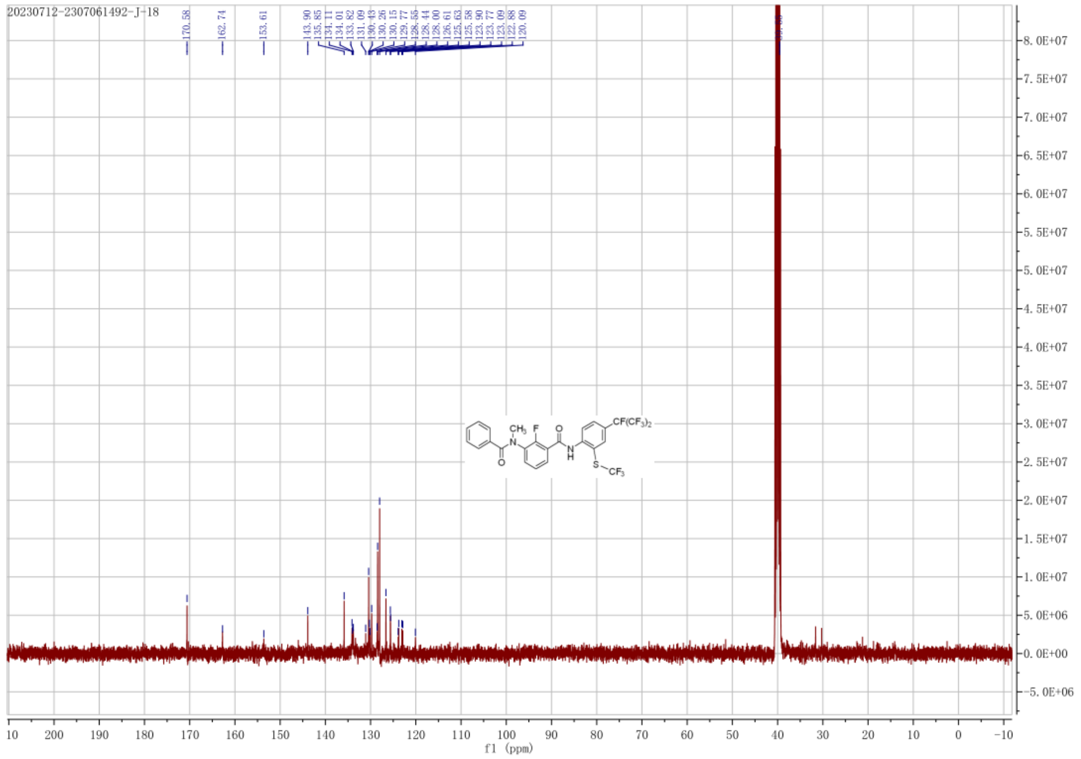

Figure 5S *^{13}C NMR spectrum of* ***A-2***

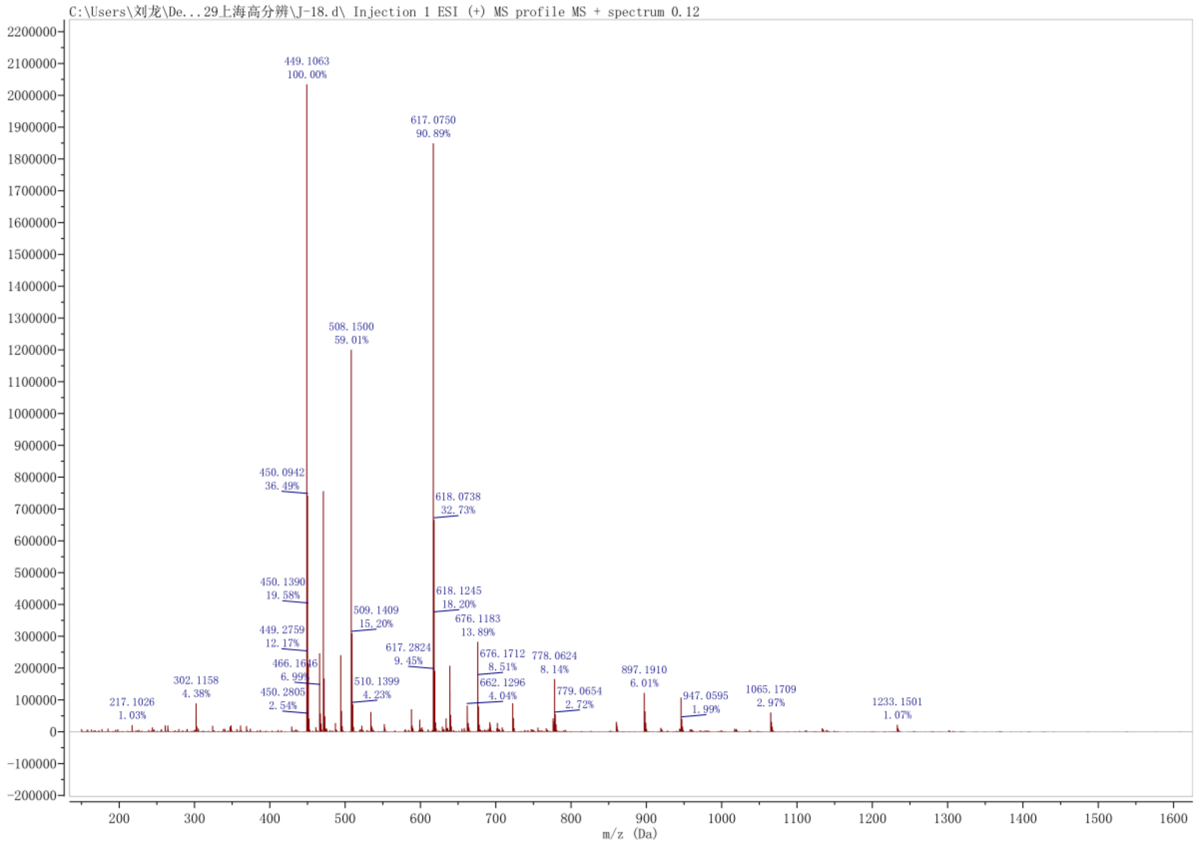

Figure 6S *HRMS spectrum of* ***A-2***

A-3

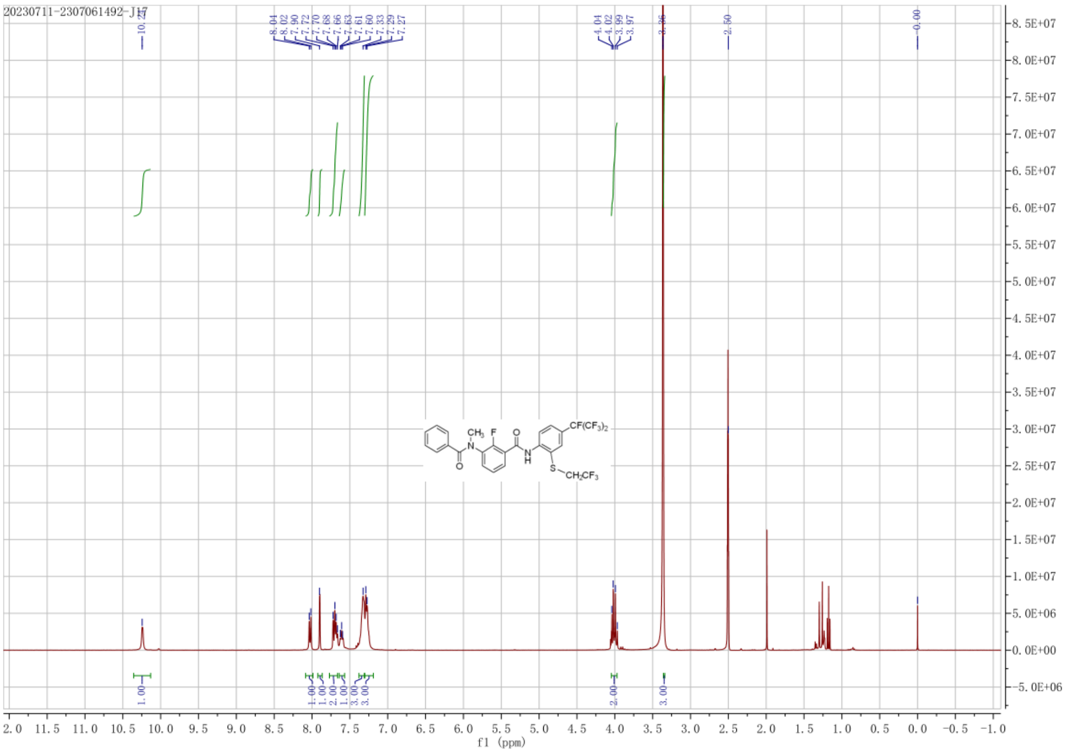

Figure 7S *^{1}H NMR spectrum of* ***A-3***

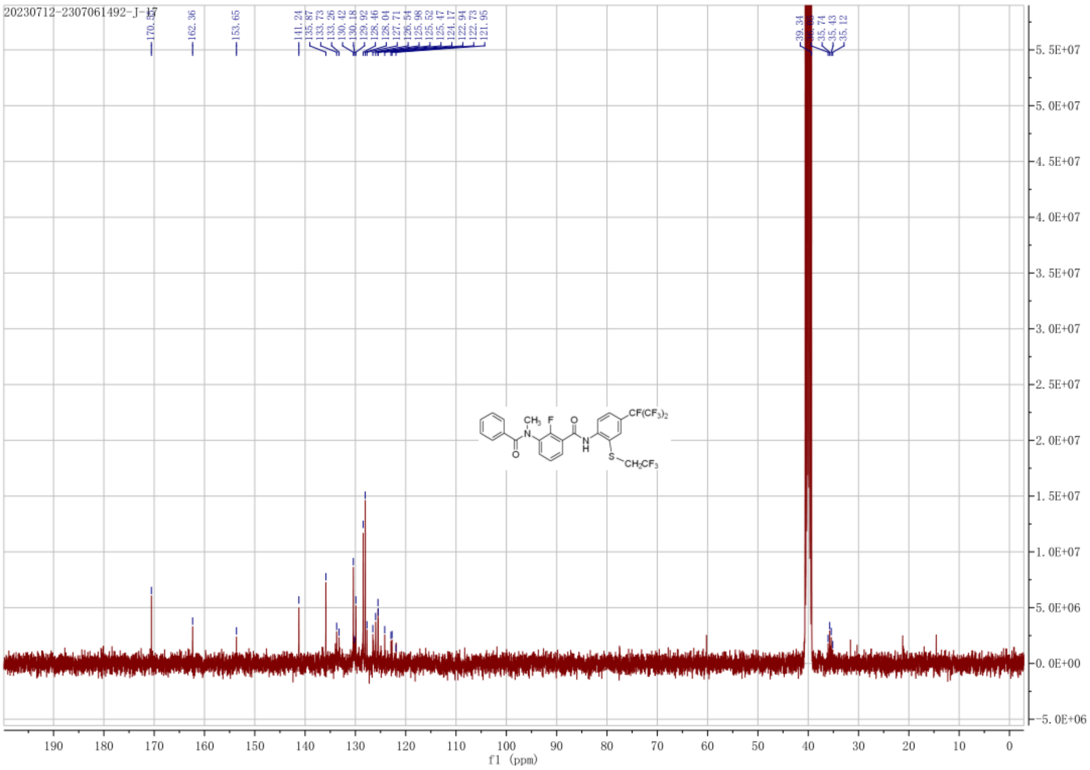

Figure 8S *^{13}C NMR spectrum of* ***A-3***

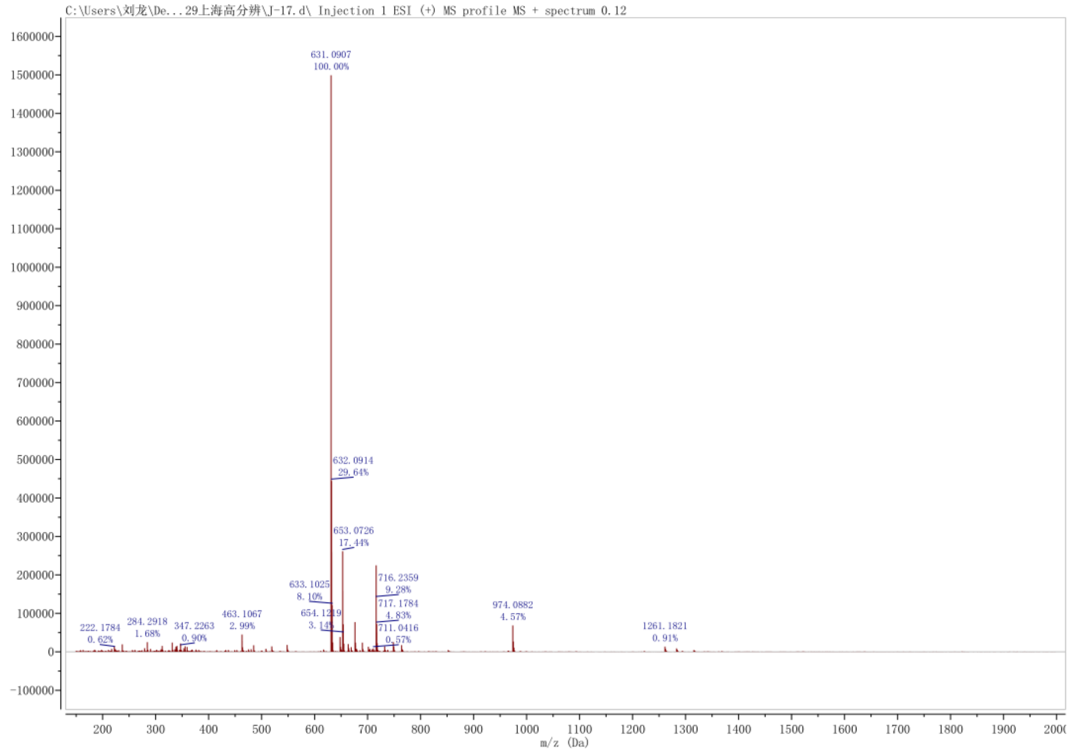

Figure 9S HRMS spectrum of ***A-3***

B-1

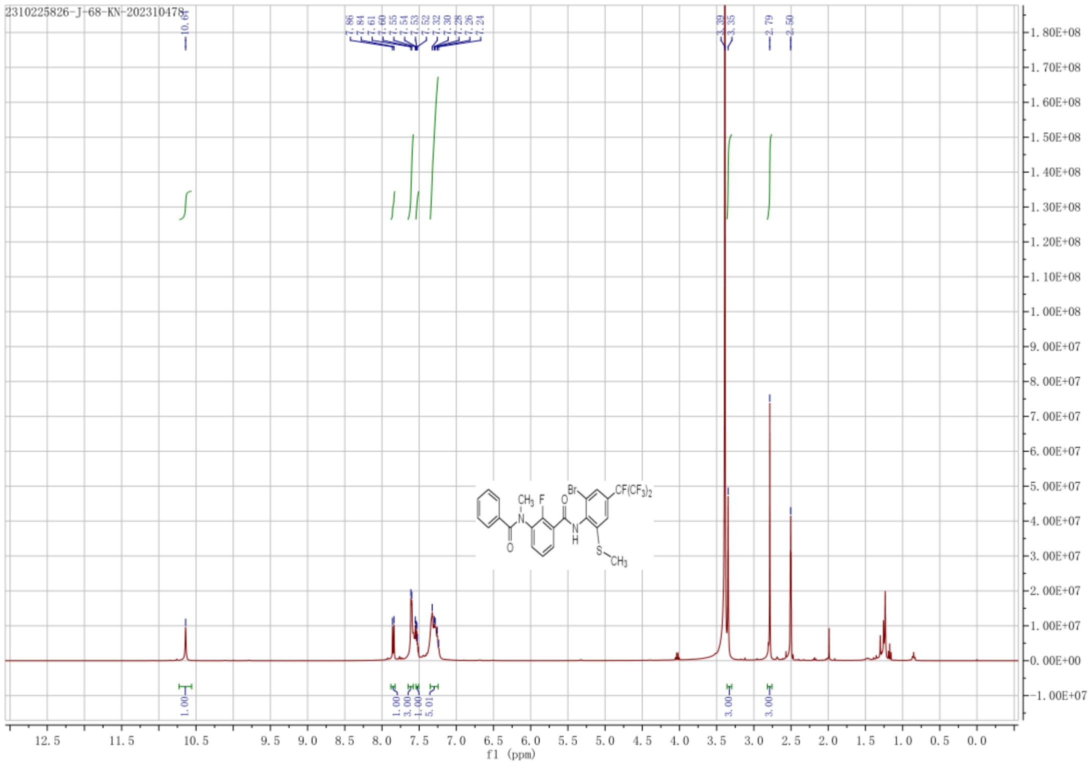

Figure 10S ^{1}H NMR spectrum of ***B-1***

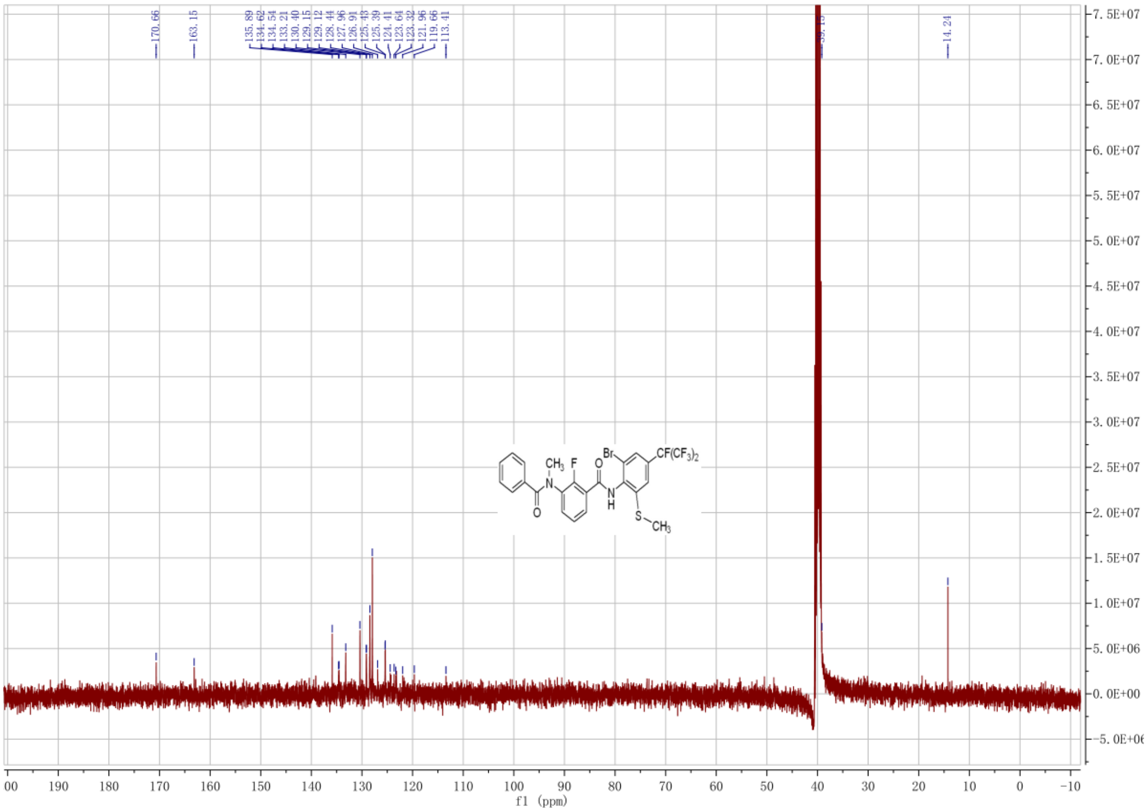

Figure 11S ^{13}C NMR spectrum of **B-1**

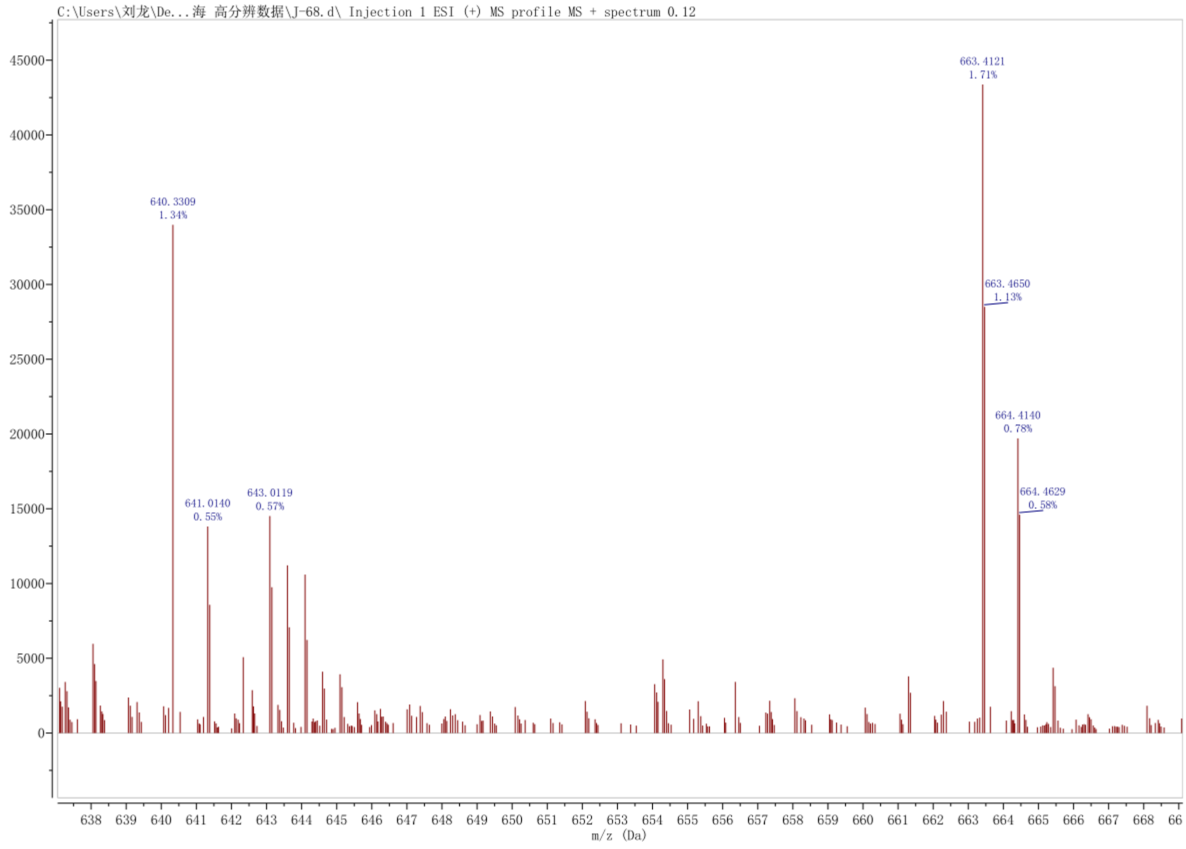

Figure 12S HRMS spectrum of **B-1**

B-2

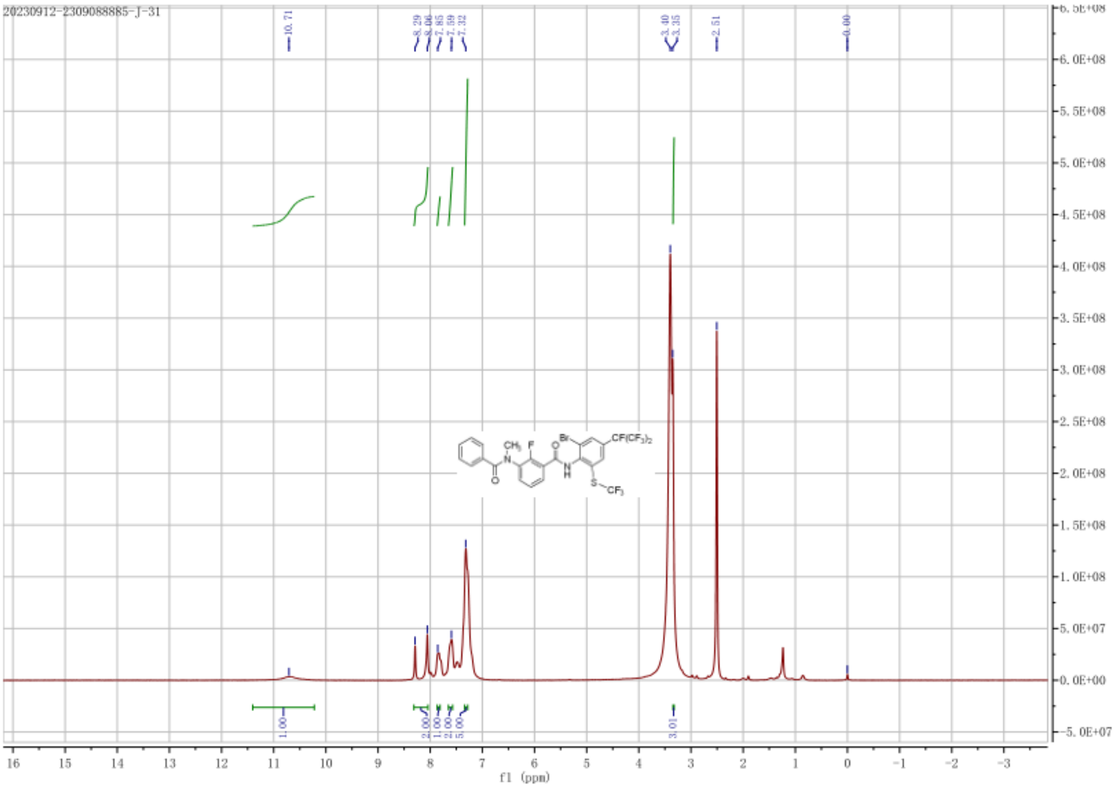

Figure 13S** ^{1}H NMR spectrum of **B-2

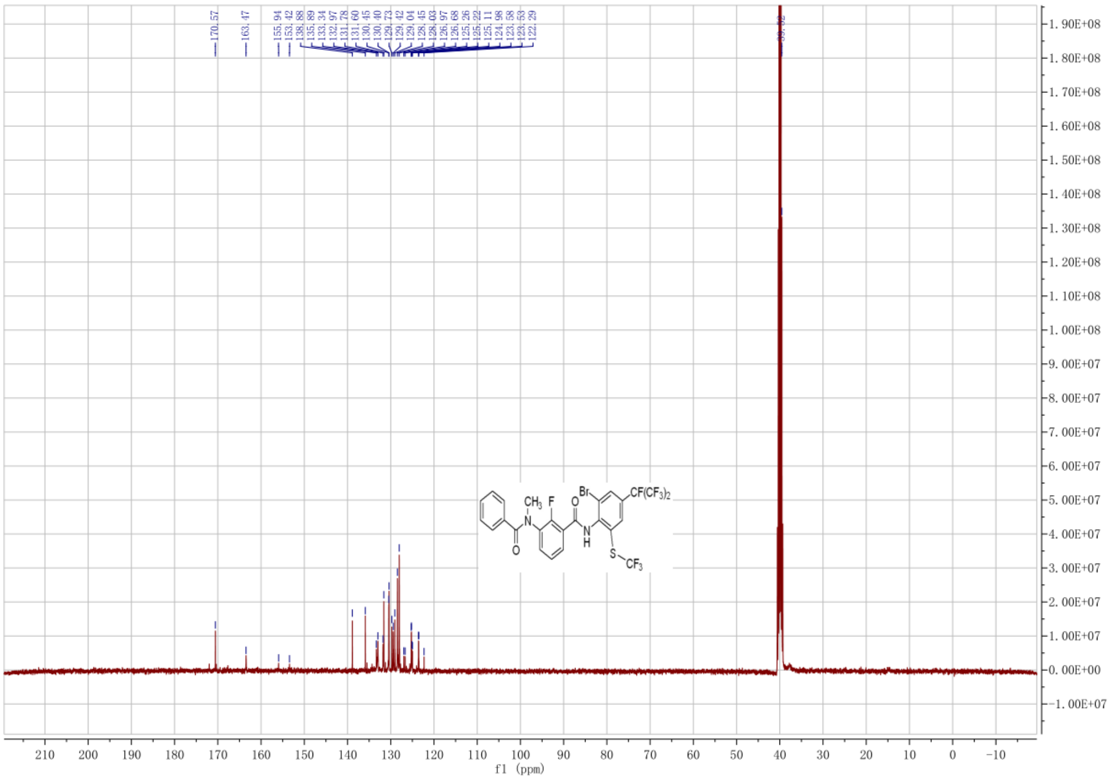

Figure 14S** ^{13}C NMR spectrum of **B-2

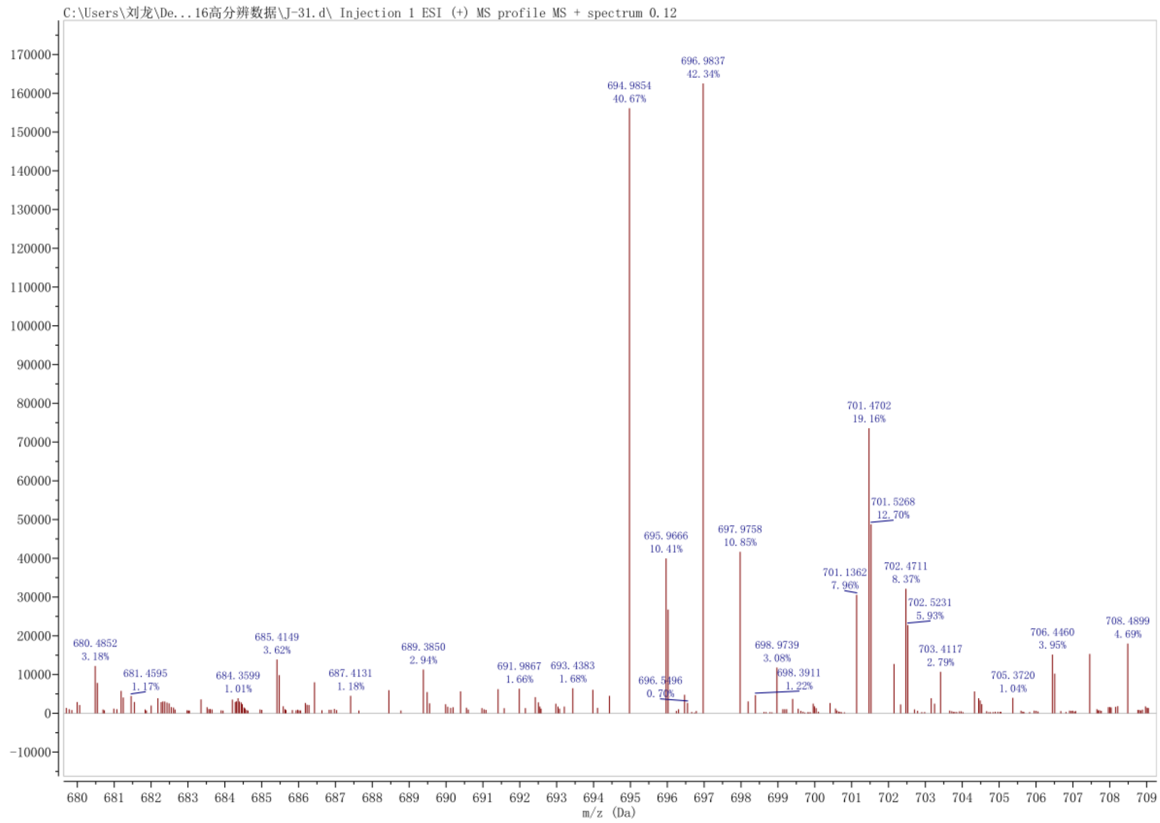

Figure 15S** HRMS spectrum of **B-2

B-3

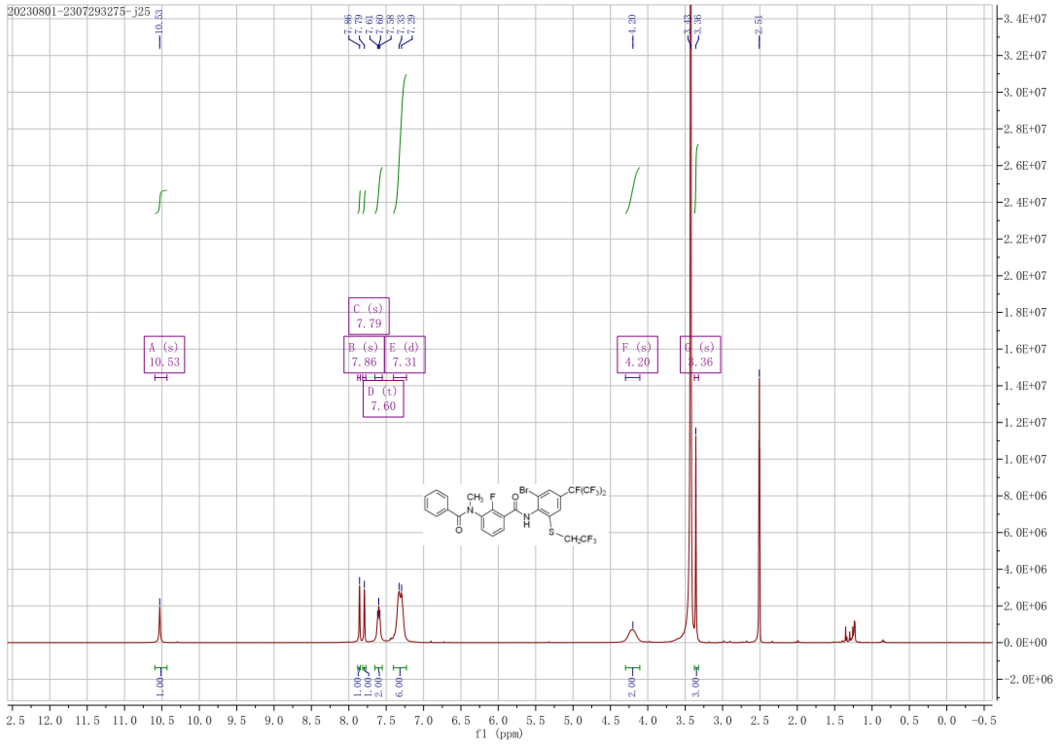

Figure 16S** ^{1}H NMR spectrum of **B-3

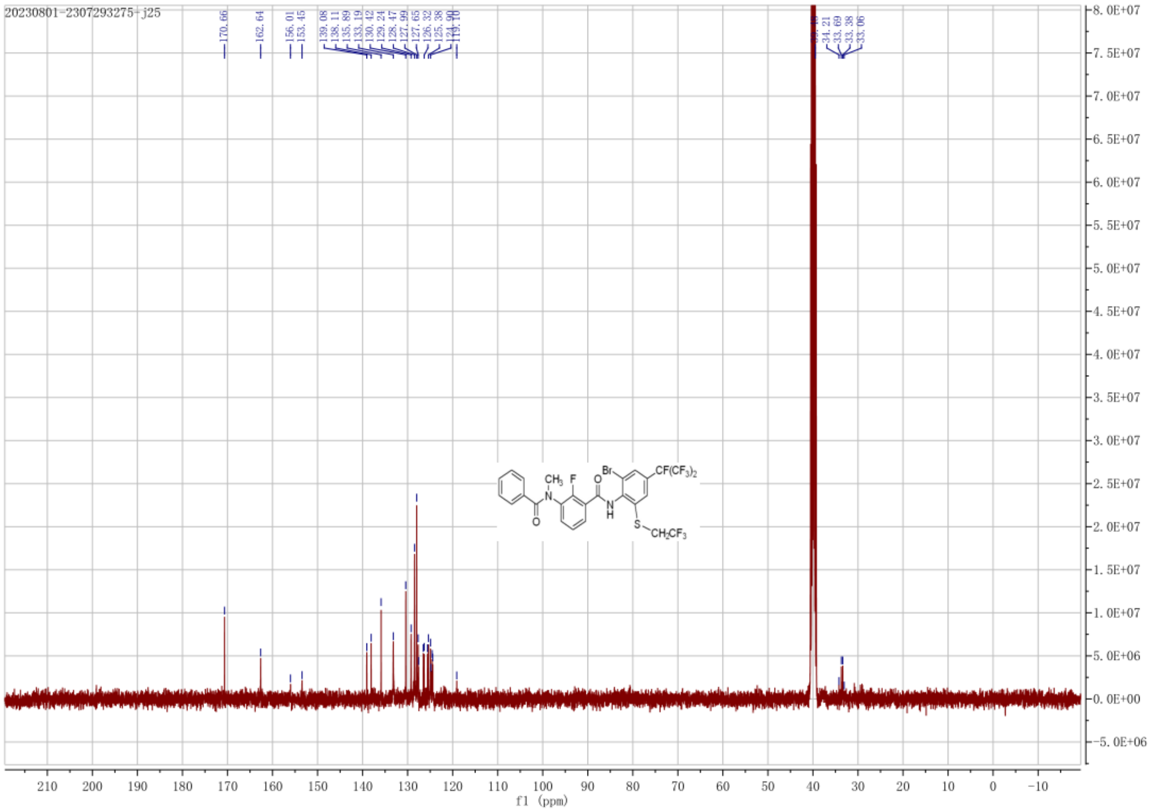

Figure 17S ^{13}C *NMR spectrum of **B-3***

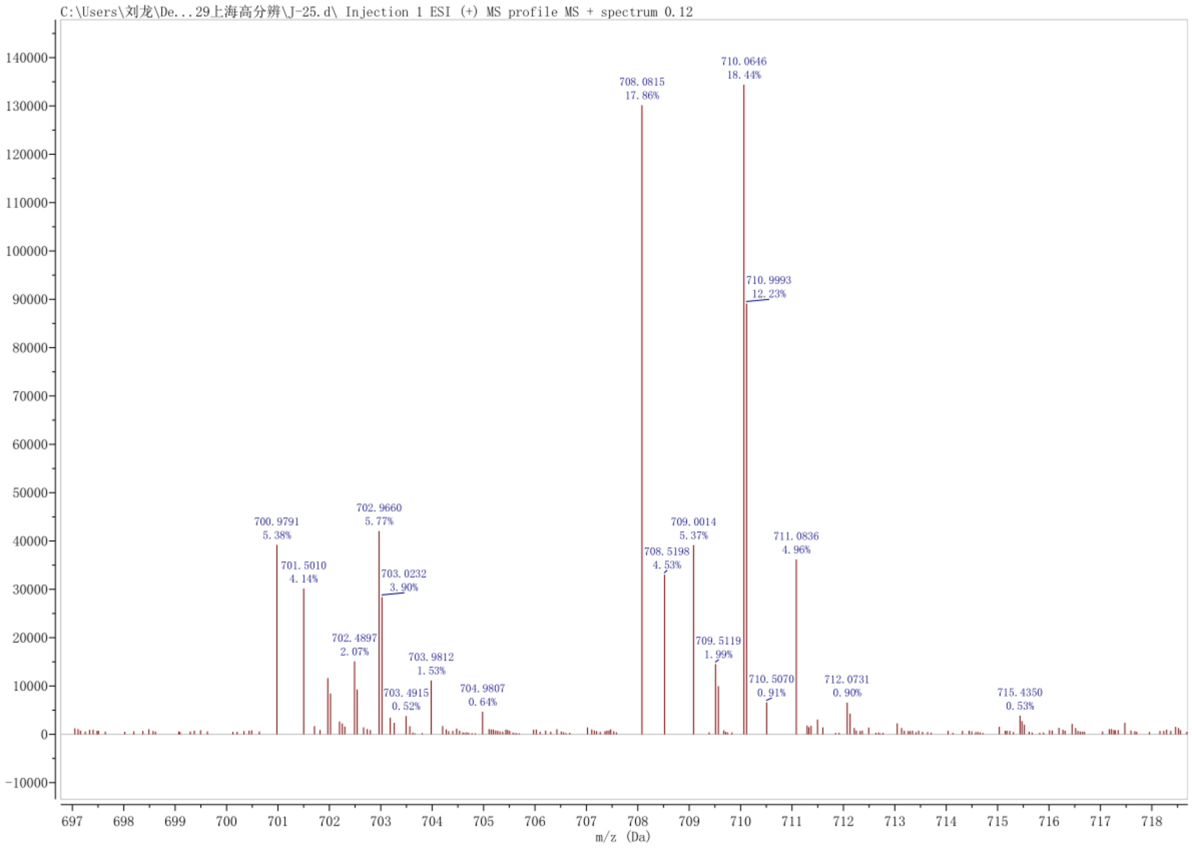

Figure 18S** HRMS spectrum of **B-3

C-1

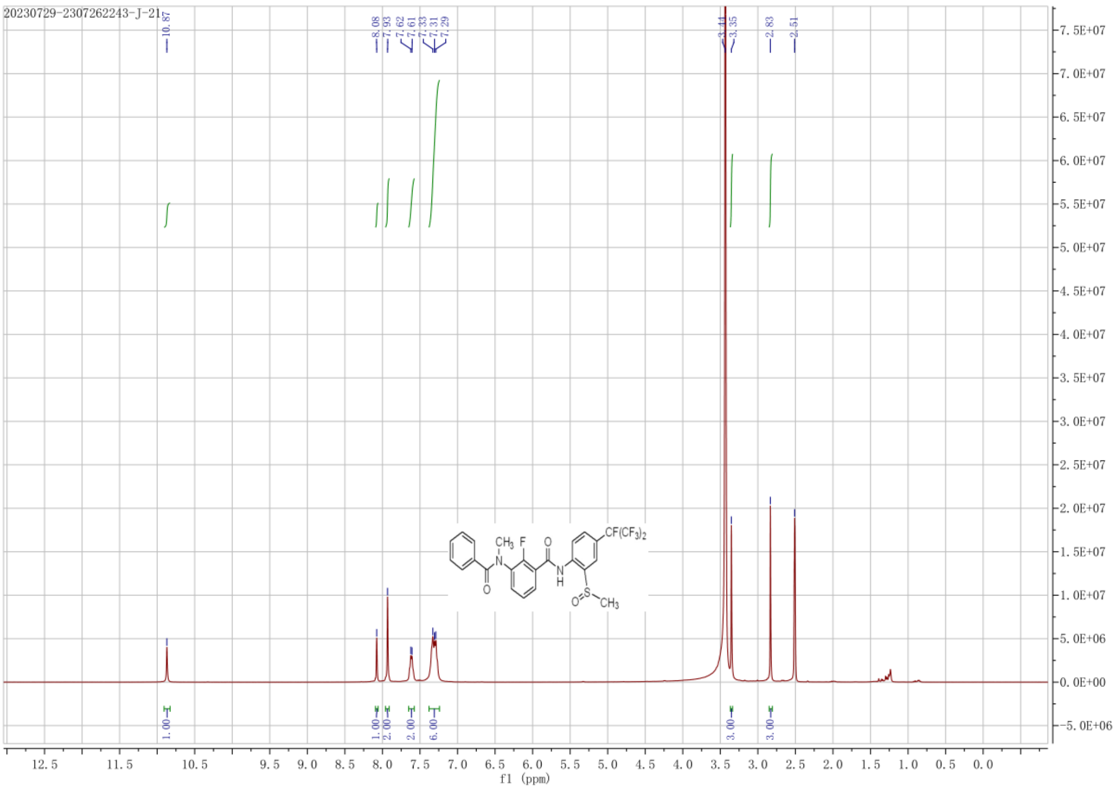

Figure 19S** ^{1}H NMR spectrum of **C-1

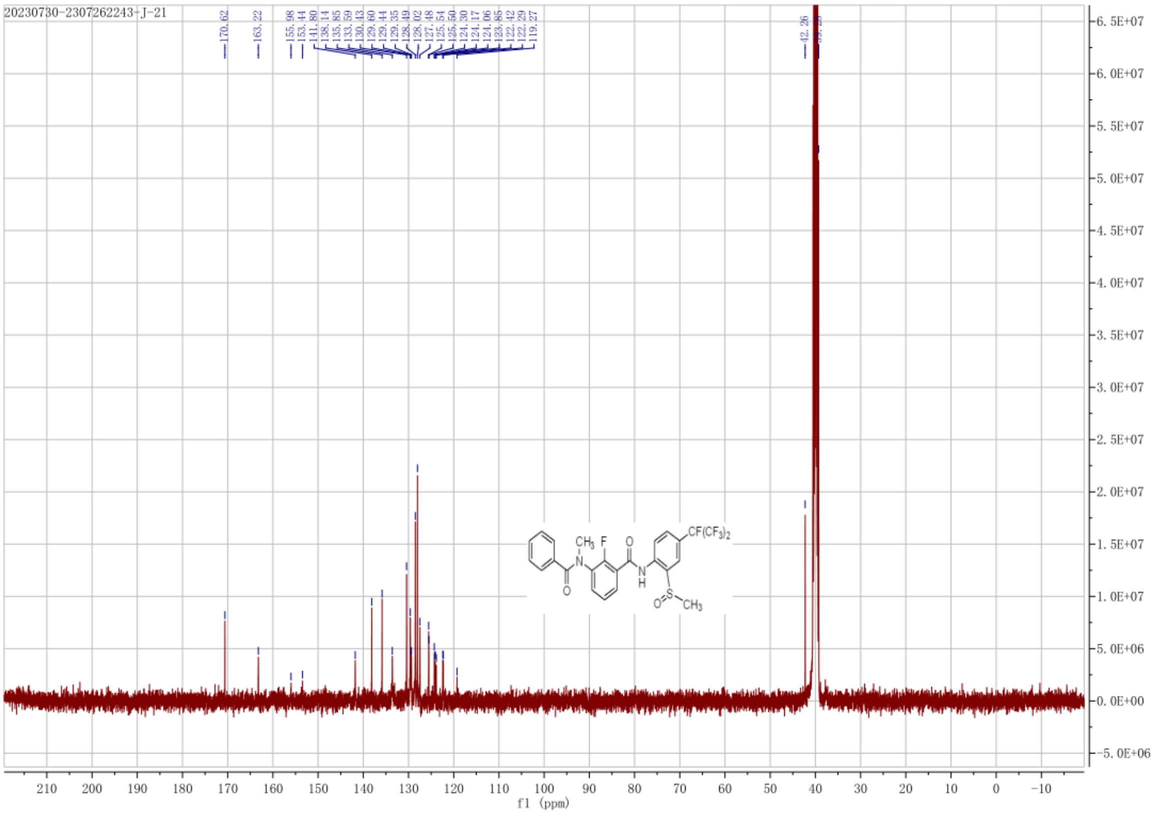

Figure 20S** ^{13}C NMR spectrum of **C-1

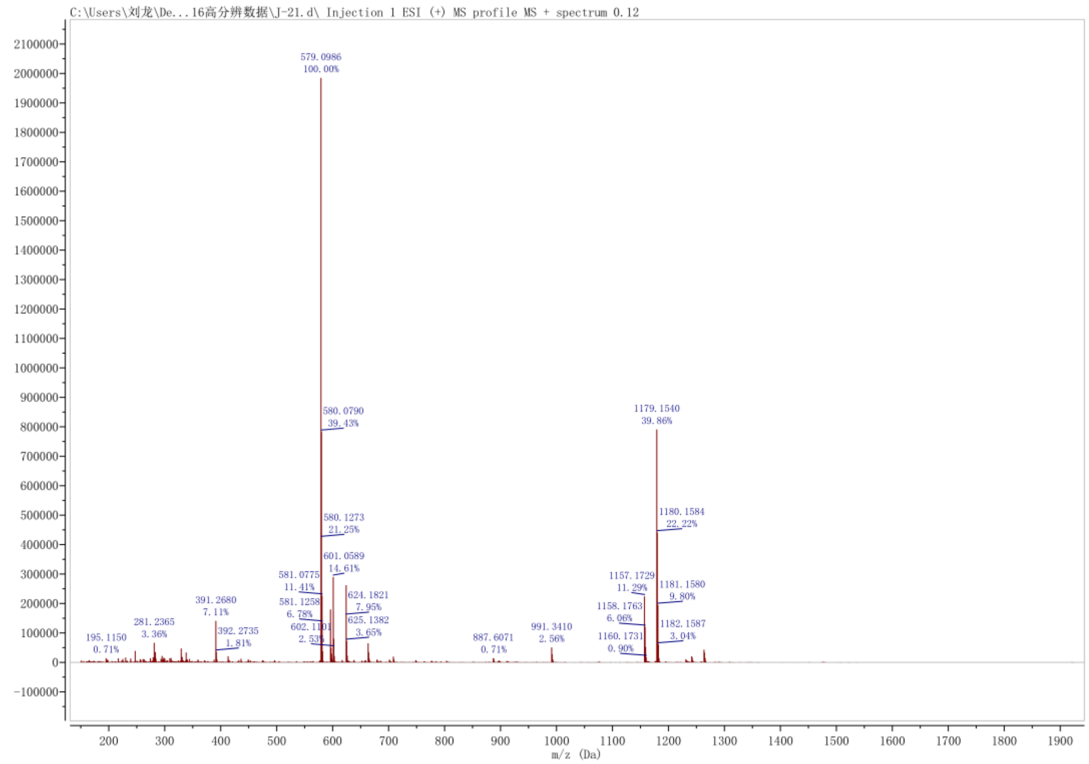

Figure 21S** HRMS spectrum of **C-1

C-2

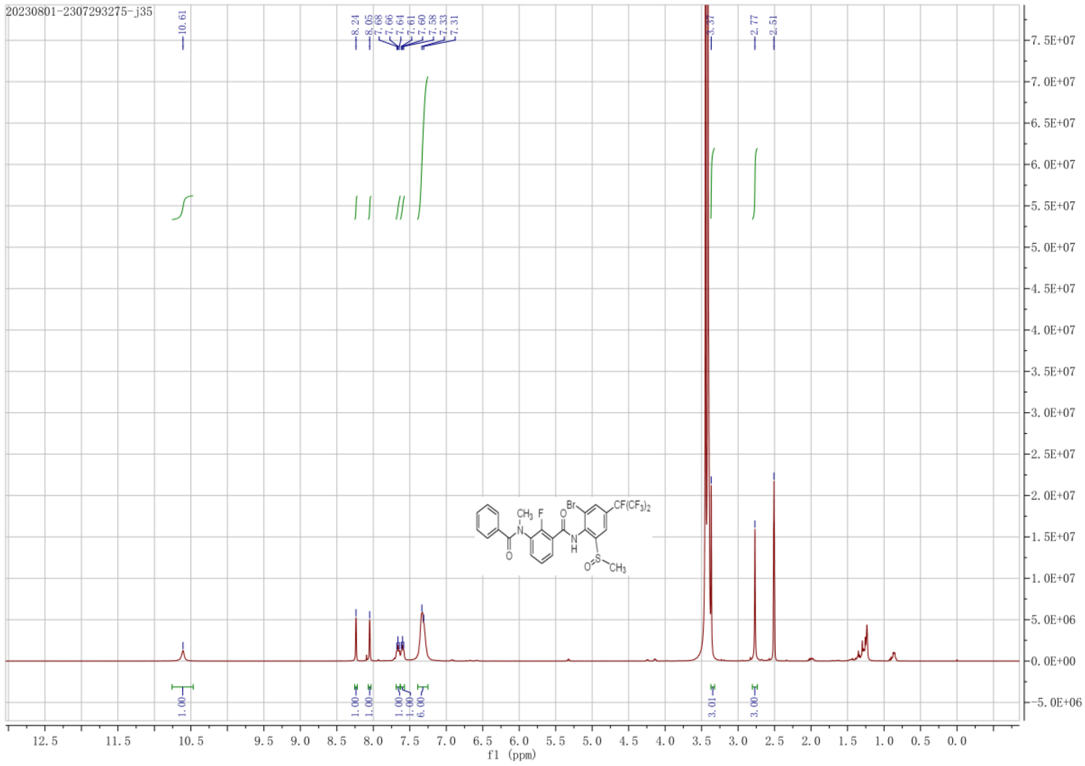

Figure 22S** ^{1}H NMR spectrum of **C-2

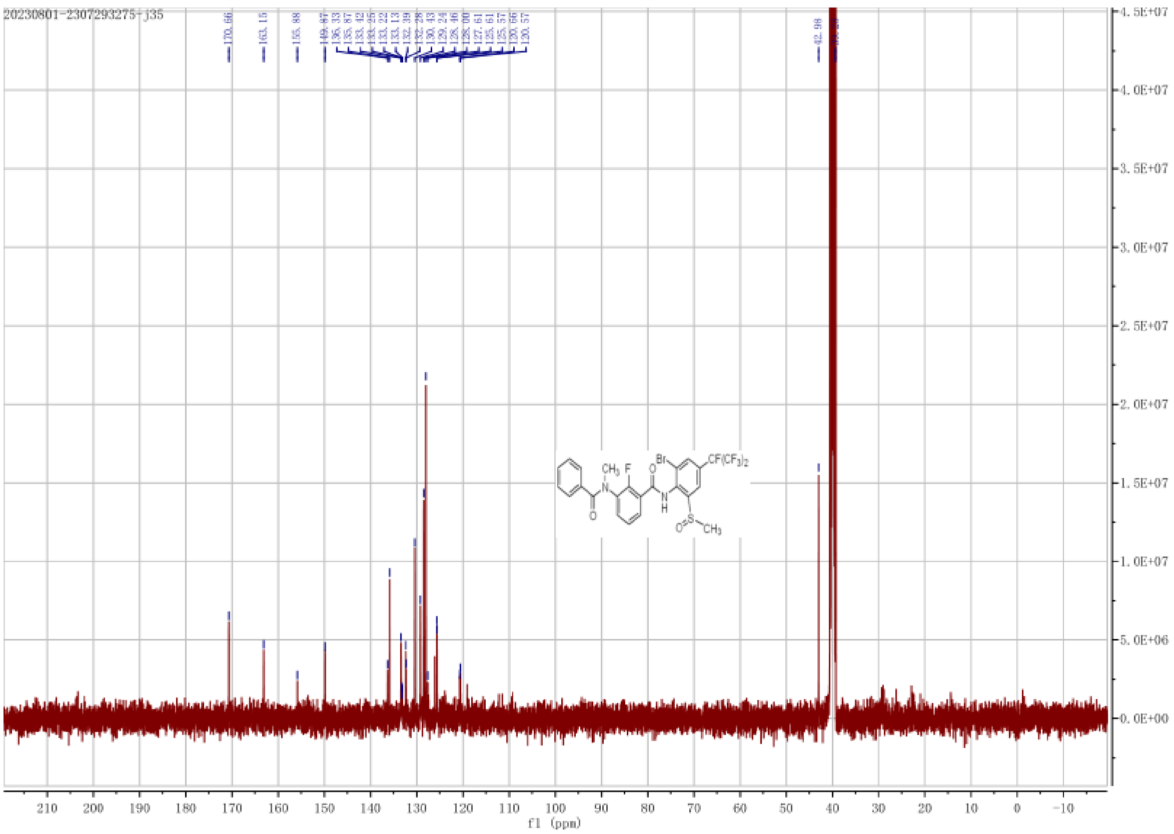

Figure 23S *^{13}C NMR spectrum of* ***C-2***

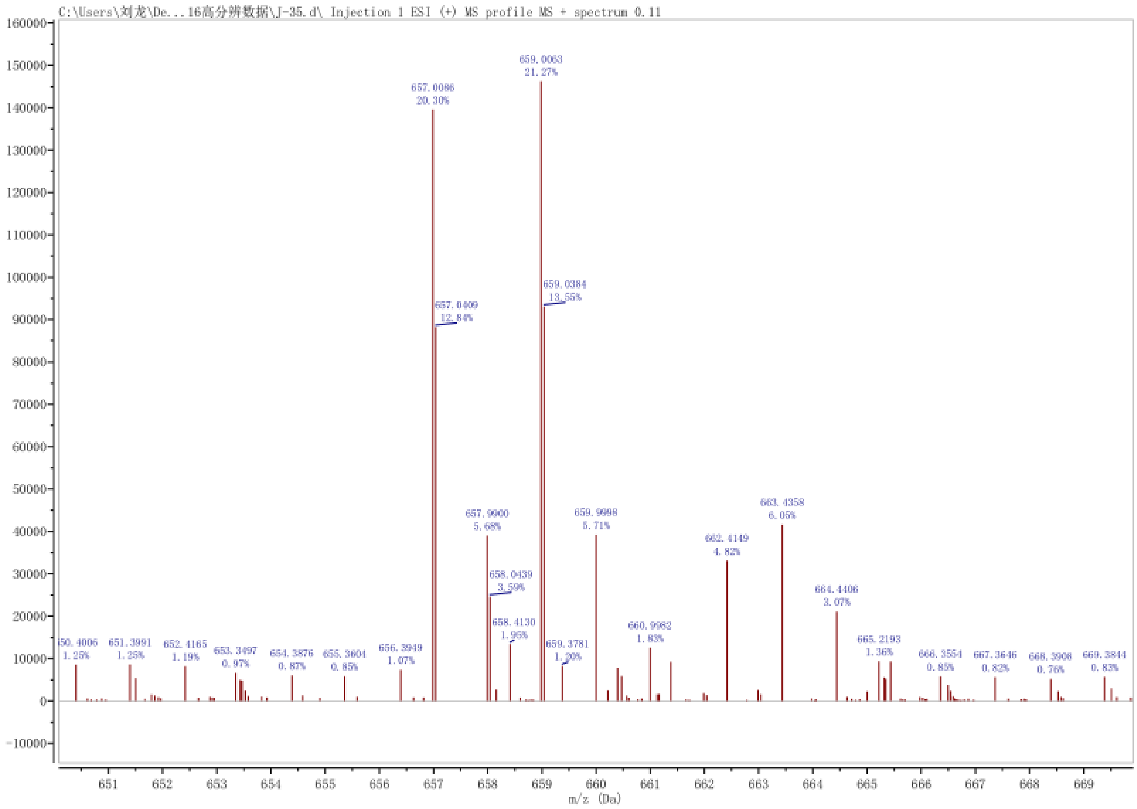

Figure24 S *HRMS spectrum of* ***C-2***

C-3

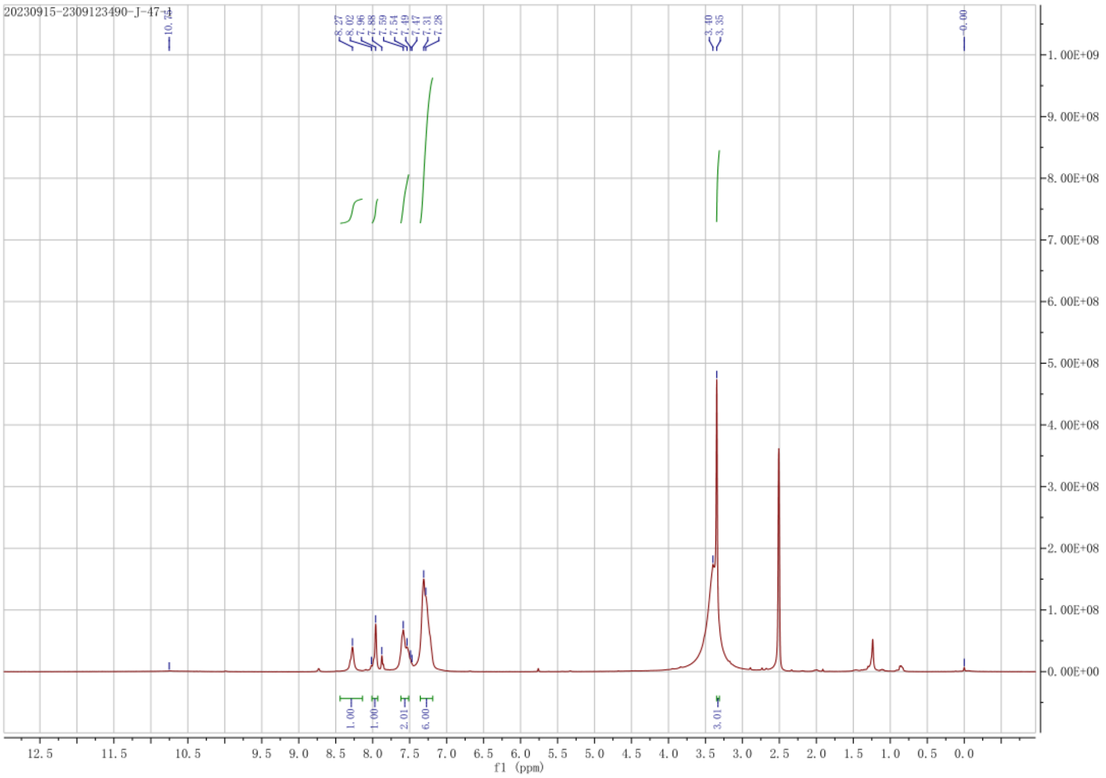

Figure 25S** ^{1}H NMR spectrum of **C-3

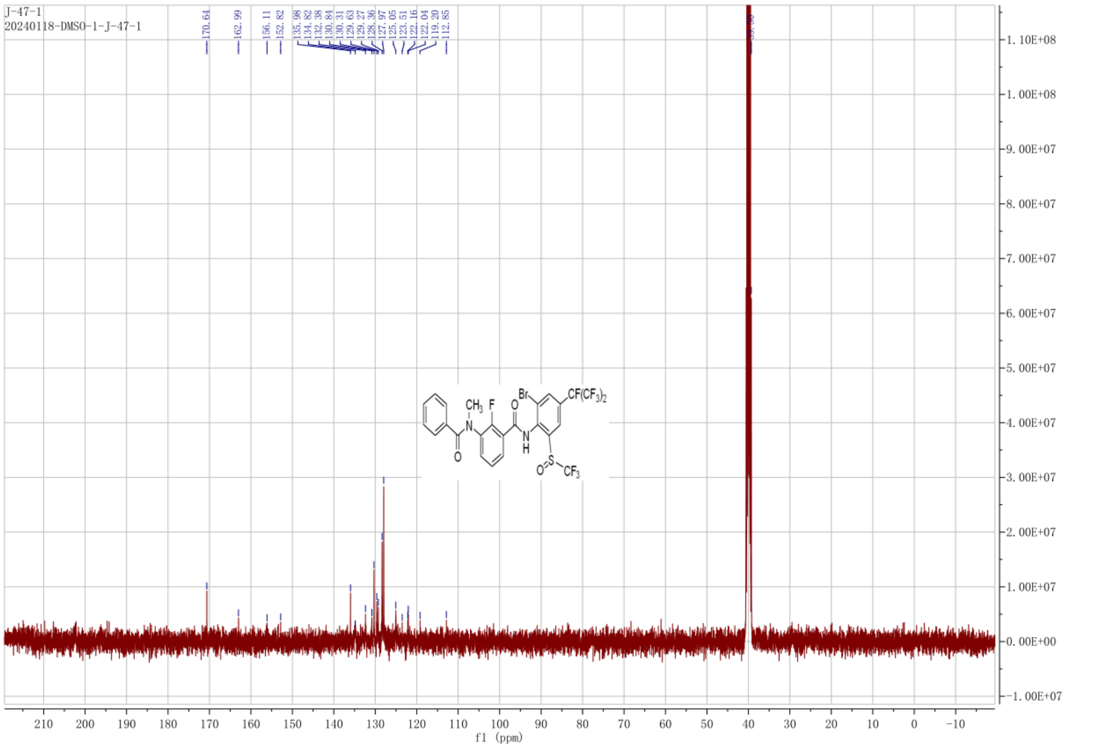

Figure 26S** ^{13}C NMR spectrum of **C-3

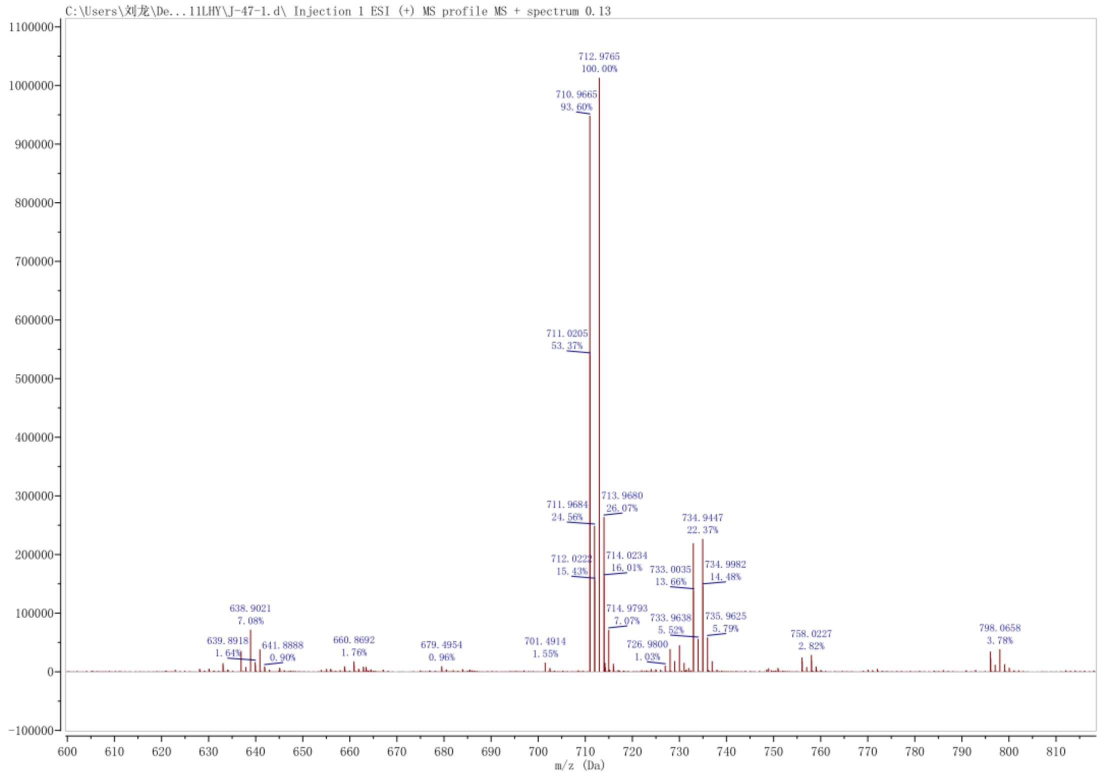

Figure 27S HRMS spectrum of ***C-3***

C-4

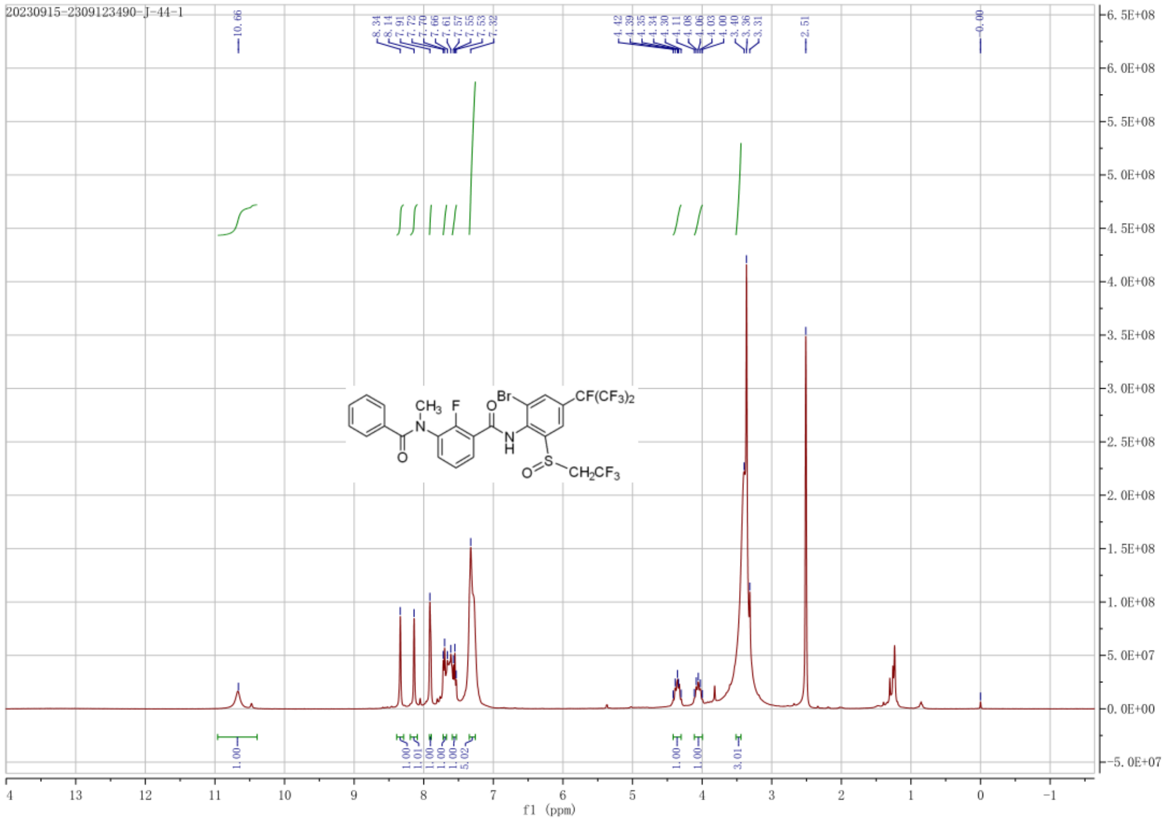

Figure 28S ^{1}H *NMR spectrum of* ***C-4***

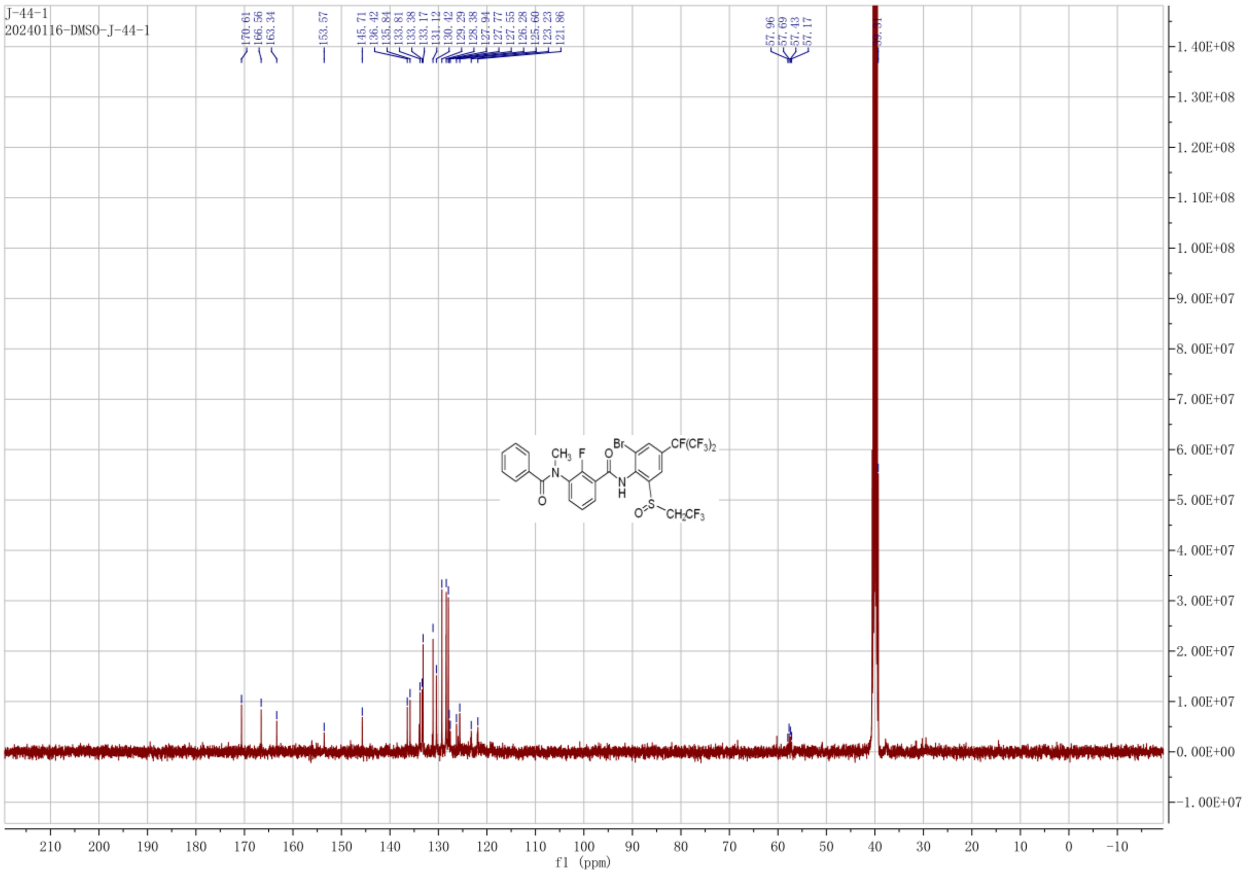

Figure 29S** ^{13}C NMR spectrum of **C-4

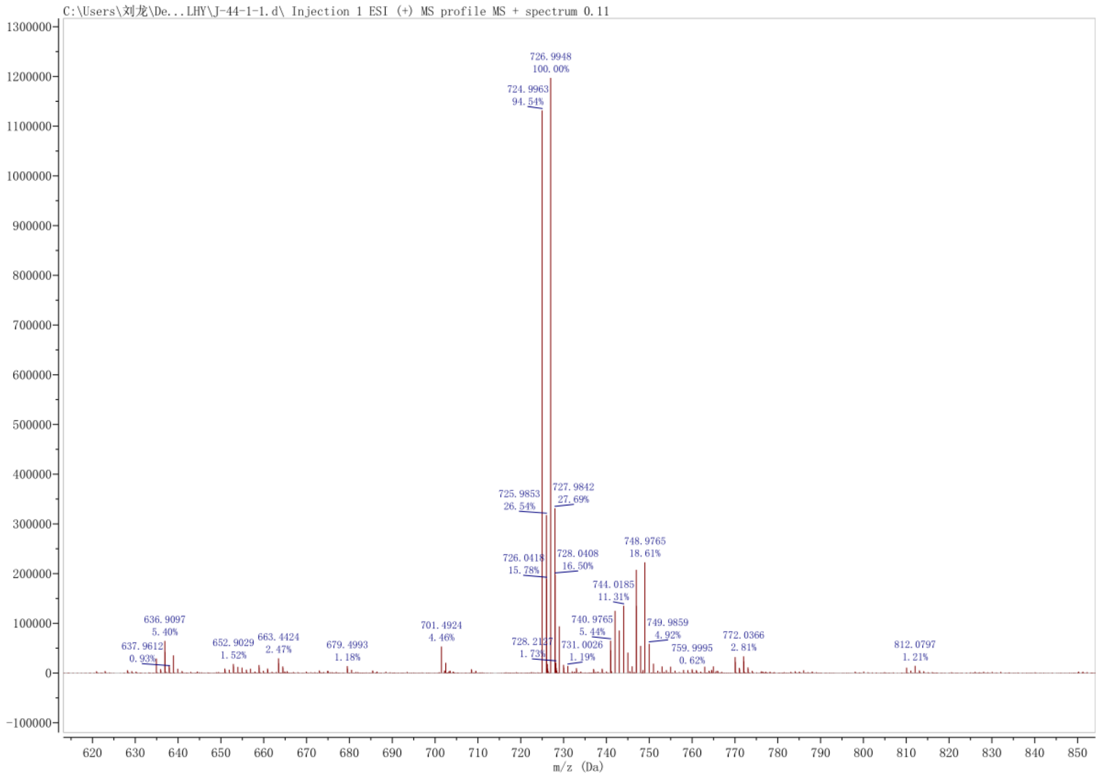

Figure 30S** HRMS spectrum of **C-4

D-1

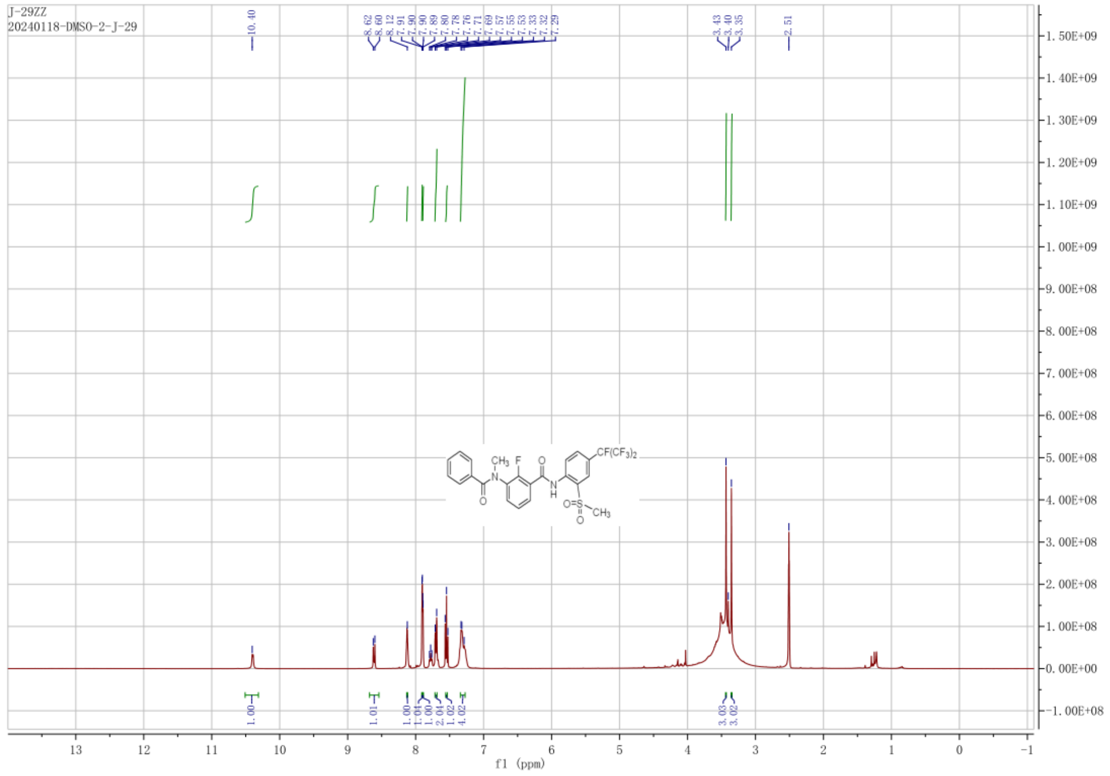

Figure 31S *^{1}H NMR spectrum of* ***D-1***

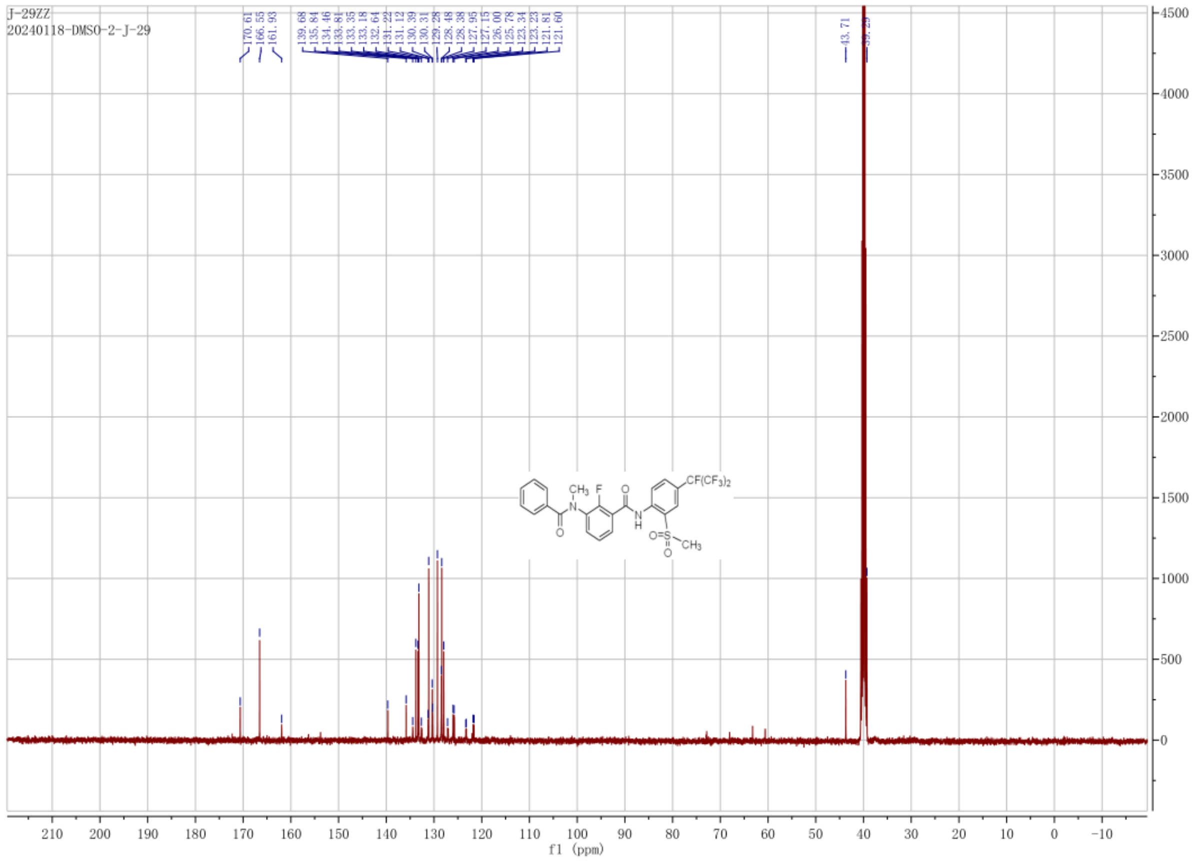

Figure 32S *^{13}C NMR spectrum of* ***D-1***

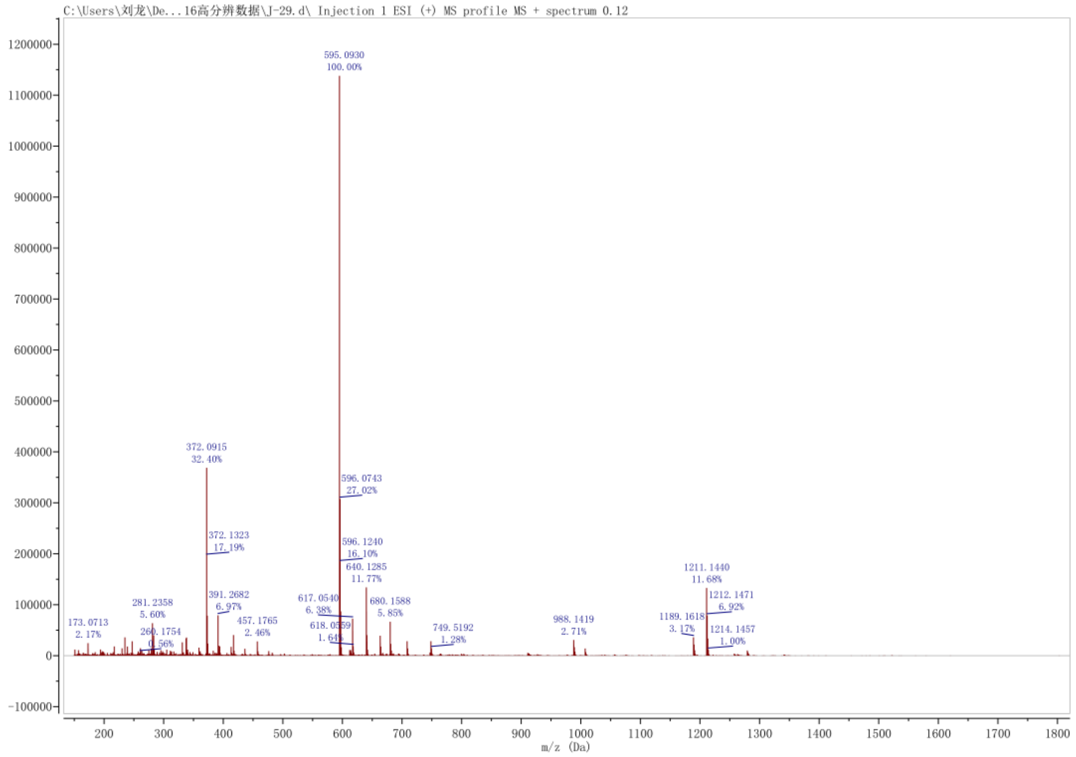

Figure 33S** HRMS spectrum of **D-1

D-2

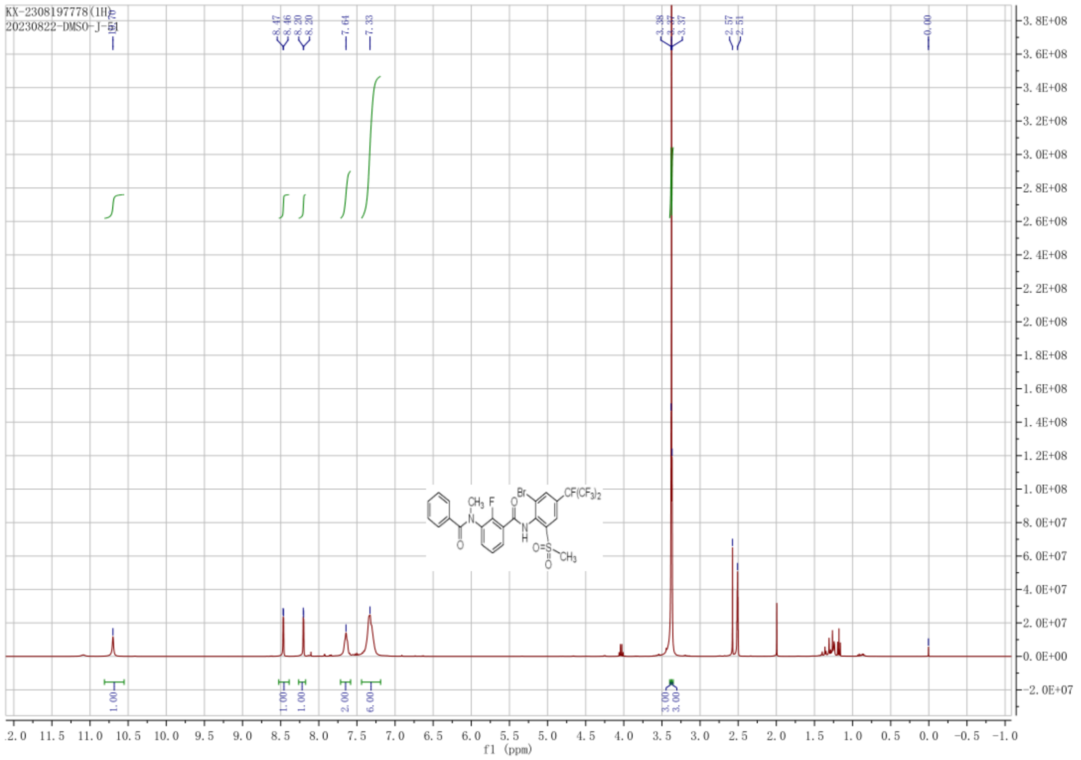

Figure 34S** ^{1}H NMR spectrum of **D-2

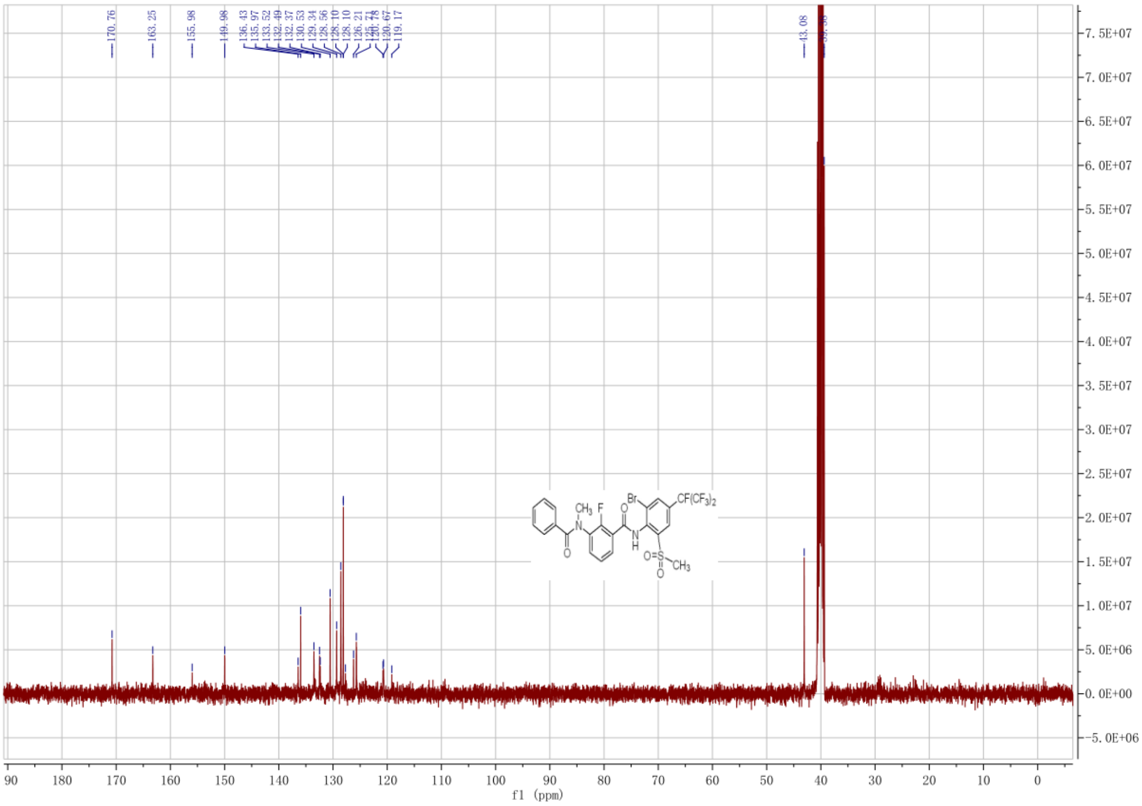

Figure 35S *^{13}C NMR spectrum of* ***D-2***

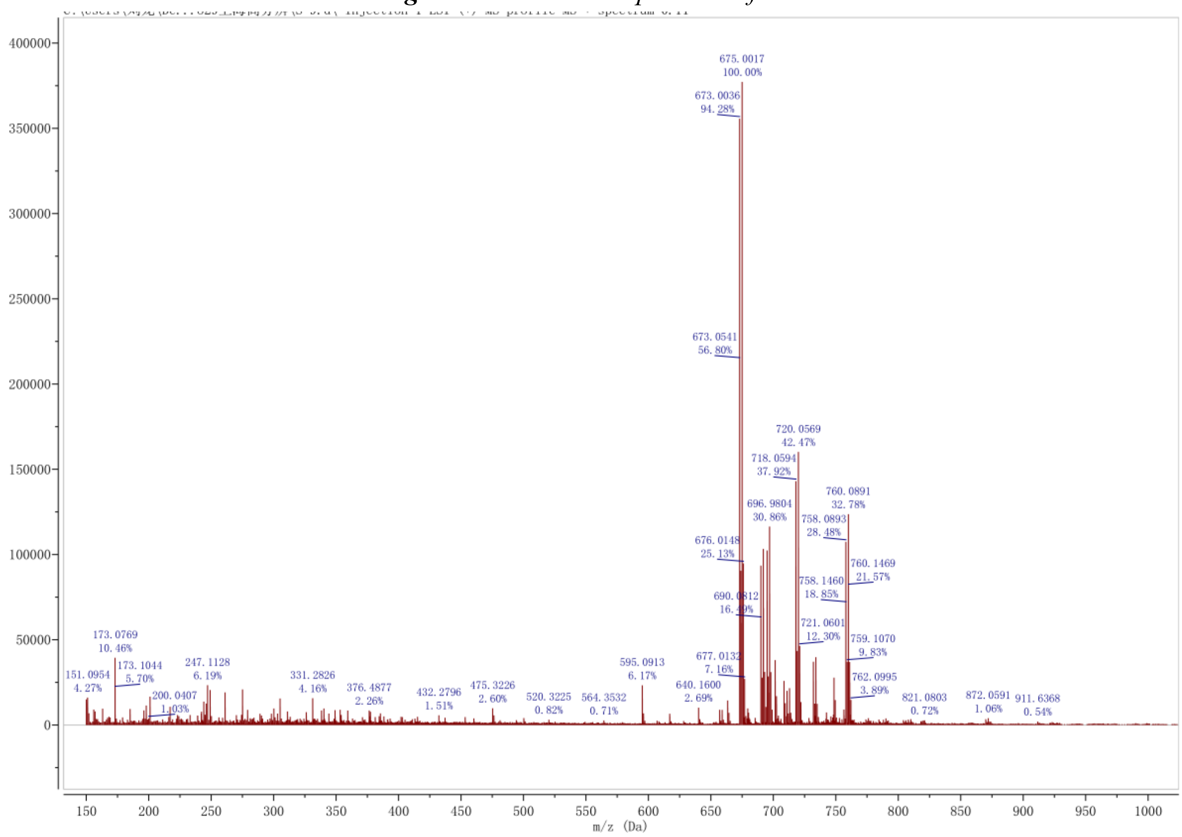

Figure 36S *HRMS spectrum of* ***D-2***
